# Transient RNA structures cause aberrant influenza virus replication and innate immune activation

**DOI:** 10.1126/sciadv.abp8655

**Published:** 2022-09-09

**Authors:** Hollie French, Emmanuelle Pitré, Michael S. Oade, Elizaveta Elshina, Karishma Bisht, Alannah King, David L.V. Bauer, Aartjan J.W. te Velthuis

**Affiliations:** ^1^ University of Cambridge, Department of Pathology, Addenbrooke’s Hospital, Cambridge CB2 2QQ, UK.; ^2^ Lewis Thomas Laboratory, Department of Molecular Biology, Princeton University, Princeton, NJ 08544, USA.; ^3^ RNA Virus Replication Laboratory, The Francis Crick Institute, 1 Midland Road, London NW1 1AT, UK.

## Abstract

During infection, the influenza A virus RNA polymerase produces both full-length and aberrant RNA molecules, such as defective viral genomes (DVGs) and mini viral RNAs (mvRNAs). Subsequent innate immune activation involves the binding of host pathogen receptor retinoic acid–inducible gene I (RIG-I) to viral RNAs. However, it is not clear what factors determine which influenza A virus RNAs are RIG-I agonists. Here, we provide evidence that RNA structures, called template loops (t-loops), stall the viral RNA polymerase and contribute to innate immune activation by mvRNAs during influenza A virus infection. Impairment of replication by t-loops depends on the formation of an RNA duplex near the template entry and exit channels of the RNA polymerase, and this effect is enhanced by mutation of the template exit path from the RNA polymerase active site. Overall, these findings are suggestive of a mechanism involving polymerase stalling that links aberrant viral replication to the activation of the innate immune response.

## Teaser

Inefficient replication of short RNA molecules produced during influenza A virus infection leads to innate immune activation.

## INTRODUCTION

Influenza A viruses (IAVs) are important human pathogens that generally cause a mild to moderately severe respiratory disease. A range of viral, host, and bacterial factors can influence the outcome of infections with IAV (*1*, *2*). One important factor is the activation of host protein retinoic acid–inducible gene I (RIG-I) by double-stranded 5′ di- or triphosphorylated RNA (*3*, *4*). Activated RIG-I translocates to mitochondria and triggers oligomerization of mitochondrial antiviral signaling (MAVS) protein and subsequent phosphorylation of interferon regulatory factor 3 (IRF3) and nuclear factor κB (*5*, *6*), leading to the expression of innate immune genes, including interferon-β (IFN-β) and IFN-λ (*6*). Innate immune gene expression typically leads to a protective antiviral state but results in an overproduction of cytokines and chemokines when dysregulated. This phenomenon underlies the lethal pathology of infections with the 1918 H1N1 pandemic or the highly pathogenic avian IAV (*7*, *8*). Various viral and host factors have been implicated in causing immunopathology, including the products of aberrant viral replication (*9*–*11*).

During an IAV infection, the virus introduces eight ribonucleoproteins (RNPs) into the host cell nucleus. These RNPs consist of oligomeric viral nucleoprotein (NP), a copy of the viral RNA-dependent RNA polymerase, and one of the eight segments of single-stranded negative-sense viral RNA (vRNA) that make up the viral genome (*12*). The vRNA segments range from 890 to 2341 nucleotides (nt) in length, but all contain conserved 5′ triphosphorylated, partially complementary 5′ and 3′ termini (*12*). These termini serve not only as promoter for the RNA polymerase but also as agonist of RIG-I (*13*, *14*). In the context of an RNP, the termini are bound by the RNA polymerase subunits protein basic 1 (PB1), PB2, and protein acidic (PA) (*15*), and during viral replication, a second RNA polymerase is recruited to the RNP to encapsidate nascent RNA (*16*, *17*). It has been hypothesized that binding of the RNA polymerase to the vRNA termini may reduce RIG-I binding to the vRNA segments, and it is not clear when or where RIG-I gains access to vRNAs (*9*).

In addition to full-length vRNA and complementary RNA (cRNA) molecules, the RNA polymerase can produce aberrant RNAs that are shorter than the vRNA or cRNA template from which they derive. These aberrant RNAs include defective viral genomes (DVGs) (*18*) and mini viral RNAs (mvRNAs) (*9*), which contain internal deletions between the conserved 5′ and 3′ termini (*9*, *19*–*22*). Both DVGs and mvRNAs can bind RIG-I and activate innate immune responses, but only DVGs require viral NP during viral replication, while mvRNAs do not (*9*, *11*). It is presently not fully understood what determines the ability of DVGs and mvRNAs to activate RIG-I or how they are made. The RNA polymerases of the highly pathogenic avian H5N1 and the pandemic 1918 H1N1 IAV produce higher mvRNA levels than the RNA polymerase of the laboratory-adapted H1N1 IAV (*9*), suggesting that there is a correlation between adaptive mutations in the RNA polymerase, mvRNA production, and innate immune activation in infections with highly pathogenic IAV.

**Fig. 1.**
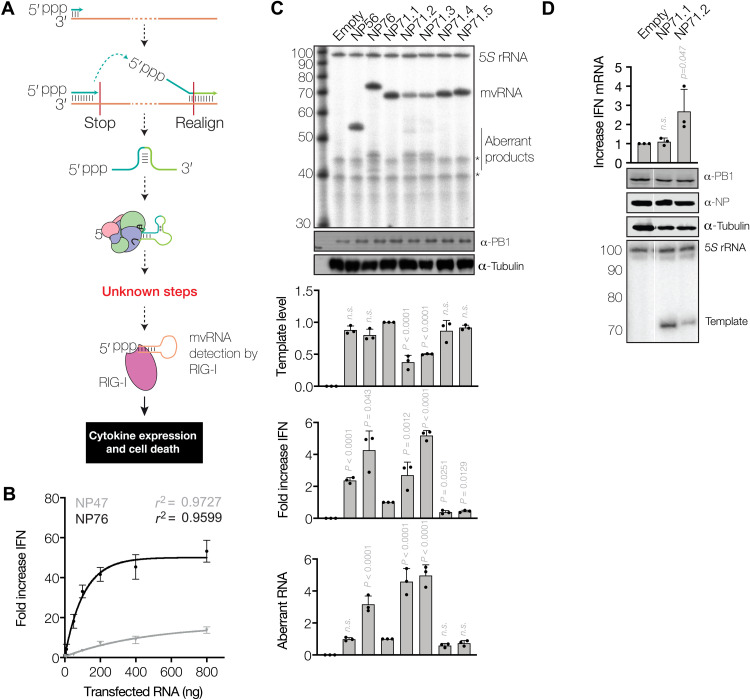
Sequence-dependent reduction of mvRNA replication and induction of IFN-β promoter activation. (**A**) Schematic of mvRNA formation via intramolecular template switching. This process has the potential to create novel RNA structures. Produced mvRNAs are bound by RIG-I, leading to the expression of innate immune responses. ppp, triphosphate. (**B**) IFN-β promoter activation following transfection of in vitro transcribed segment 5 mvRNAs of 47 or 76 nt in length. (**C**) Replication of model mvRNAs in HEK293T cells by the influenza virus A/WSN/33 (H1N1) RNA polymerase. RNA levels were analyzed by primer extension, and the ability of mvRNA replication to induce IFN-β promoter activity was analyzed using a luciferase-based IFN-β reporter assay. Nonspecific primer extension signals in (C) are indicated with *. (**D**) RT-qPCR analysis of IFN-β mRNA levels. Data from three biological repeats are shown. n.s., not significant.

We here aimed to understand the role of mvRNAs in innate immune activation in more detail. mvRNAs are generated, in part, via a copy-choice mechanism that results in the loss of an internal genome segment sequence (*9*), similar to what has been observed for DVGs (Fig. 1B) (*23*–*25*). As a result, RNA sequences or structures that do not normally reside side by side in the full-length genome segments are brought closer to each other in the nascent RNA, potentially resulting in the formation of novel RNA structures (Fig. 1B). Once generated, mvRNAs can be replicated by the RNA polymerase in the absence of NP (*26*). Inherently, the RNA polymerase is not impaired by RNA structures in an mvRNA template, and it can replicate and transcribe an mvRNA containing a copy of the aptamer Spinach (*27*), a highly-structured RNA capable of stabilizing the fluorophore 3,5-difluoro-4-hydroxybenzylidene imidazolinone (DFHBI) (fig. S1). However, it is not known whether other RNA secondary structures or certain sequence combinations could impair mvRNA replication or play a role in the activation of the innate immune response during IAV infection, as for instance has been observed for paramyxovirus infections (*28*). Here, we advance our previous model on the effect of mvRNAs on the innate immune response and provide evidence that mvRNAs capable of inducing innate immune responses may contain RNA structures that can reduce the activity of the IAV RNA polymerase.

## RESULTS

### Induction of IFN-β promoter activation by mvRNAs is sequence dependent

mvRNAs bind RIG-I and activate the MAVS signaling cascade (*9*, *11*), but it is unclear what determines whether an IAV mvRNA is an inducer of the innate immune response. To systematically investigate whether the sequence or secondary structure of an mvRNA can affect IAV RNA polymerase activity and innate immune activation, we engineered five segment 5–derived mvRNA templates. Each engineered mvRNA had a length of 71 nt (NP71.1 to NP71.5) but a different internal sequence (table S1). Our positive control mvRNAs were 56- and 76-nt-long mvRNAs, which we had previous constructed from segment 5 (NP56 and NP76, respectively), while our negative control mvRNA was a 47-nt-long mvRNA derived from segment 5 (NP47) that is unable to bind RIG-I and induce a strong IFN signal (*9*). To validate our test setup, we transfected increasing amounts of in vitro–transcribed NP76 into human embryonic kidney (HEK) 293T cells and found a strong increase in IFN-β promoter activity that saturated at a ~50-fold induction, while the NP47 induced a lower activity (Fig. 1B). These observations show how these RNAs differentially induce IFN-β promoter activity when they are transfected into the cytoplasm.

We subsequently validated the IFN-β promoter activation by the NP47, NP56, and NP76 mvRNA templates during replication by the IAV RNA polymerase. To this end, we transfected plasmids expressing the RNA polymerase subunits PB1, PB2, and PA; a plasmid expressing NP; and a plasmid expressing the NP76 template mvRNA into HEK293T cells. Primer extension analysis showed efficient amplification of NP47, NP56, and NP76 and the production of several smaller aberrant RNA products that were shorter than the mvRNA template in the case of NP56 and NP76 (Fig. 1C and fig. S2). We also observed IFN-β promoter activation by NP56 and NP76 but not NP47. These results thus indicate that similar to the full-length vRNA segments, mvRNAs themselves can also serve as template for aberrant RNA synthesis. Fractionation of cells in which NP76 was replicated showed that the NP76 mvRNA template was present in the nuclear, cytoplasmic, and mitochondrial fractions, whereas the aberrant RNAs produced from the NP76 template were present in the nucleus only (fig. S2). Because IAV RNA is predominantly detected in the cytoplasm of the host cell, these results suggest that the mvRNA template and not aberrant products shorter than the mvRNA template play a role in innate immune activation.

Following the characterization of our assays, we next analyzed the replication and IFN-β promoter activation by the engineered mvRNA templates and found that three of these templates were efficiently replicated (NP71.1, NP71.4, and NP71.5), while the other two (NP71.2 and NP71.3) were not (Fig. 1C). Among the engineered mvRNAs, templates that were poorly replicated showed higher IFN-β promoter activity and aberrant RNA synthesis (i.e., the production of RNA products containing deletions relative to the template; fig. S3A) than the three mvRNA templates that were efficiently replicated (Fig. 1C). Reverse transcription quantitative polymerase chain reaction (RT-qPCR) analysis of cells replicating NP71.1 and NP71.2 confirmed that endogenous IFN-β mRNA levels were increased when NP71.2 was replicated (Fig. 1D). To confirm that the NP71.2 had the ability to induce innate immune activation during viral infection, we preexpressed NP71.1 and NP71.2 in the absence of vRNA polymerase and NP in HEK293T cells. After 24 hours, the cells were infected with three multiplicity of infection (MOI) influenza virus A/WSN/1933 (H1N1) for 8 hours. As shown in fig. S3B, preexpression of NP71.1 and NP71.2 had no effect on segment 6 replication or PB1 protein expression. In addition, we observed phosphorylation of IRF3 after preexpression of NP71.1 but not NP71.2. While this is suggestive of MAVS signaling pathway activation through replication of mvRNA by the RNA polymerase, we could not detect amplification of the exogenous mvRNAs, potentially because they need to compete with the eight endogenous vRNA templates for binding to the RNA polymerase expressed by the virus. We can thus not say whether the engineered mvRNAs affect the MAVS signaling pathway in the same way during viral infection as in our RNP reconstitution experiments.

To exclude that a differential recognition of the engineered mvRNAs by host pathogen receptors of the host cell was responsible for the observed increased IFN-β promoter activity on the NP71.2 and NP71.3 mvRNAs, we isolated total RNA from HEK293T transfections and retransfected equal amounts of these RNA extracts together with IFN-β and *Renilla* reporter plasmids into HEK293T cells. Retransfection of NP71.1 to NP71.5 showed an inverse pattern of IFN-β promoter activation in comparison to Fig. 1C, whereby abundant mvRNAs induced more IFN-β promoter activity than the least abundant mvRNAs (fig. S3C), suggesting that there is no inherent difference between the mvRNA in their ability to activate IFN-β promoter activity. Instead, these results indicated that impaired active viral replication determines whether an mvRNA will activate innate immune signaling in the context of an RNP.

To verify that the different replication efficiencies had not been the result of the effect of NP71.2 and NP71.3 on the innate immune response, we also expressed these mvRNAs and the WSN RNA polymerase in *MAVS*^−/−^ IFN::luc HEK293 cells (*29*). These *MAVS*^−/−^ cells do not express endogenous MAVS (fig. S4A), blocking any RIG-I–mediated innate immune signaling, but overexpression of a MAVS-FLAG plasmid still triggers IFN-β promoter activity, indicating that the IFN-β reporter is still functional (fig. S4B). Expression of NP71.1 to NP71.5 in the *MAVS*^−/−^ cells did not induce IFN-β promoter activity (fig. S4C). Subsequent primer extension analysis showed that the differences in replication between NP71.1 to NP71.5 had been maintained in the *MAVS*^−/−^ cells (fig. S4C), demonstrating that the differential replication efficiency is not dependent on the innate immune response.

To investigate whether the effect of the NP71.3 and NP71.4 mvRNAs was specific to the WSN polymerase, we expressed these mvRNAs alongside the pandemic H1N1 A/Brevig Mission/1/18 (abbreviated as BM18) or the highly pathogenic avian H5N1 A/duck/Fujian/01/02 (abbreviated as FJ02) RNA polymerases. We found that the BM18 and FJ02 RNA polymerases were impaired on the NP71.2 and NP71.3 mvRNA templates and triggered a stronger IFN-β promoter activity on the NP71.3 template relative to the NP71.1 template (fig. S5). We also observed that the BM18 RNA polymerase produced short aberrant RNA products, while the FJ02 RNA polymerase did not despite inducing IFN-β promoter activity (fig. S5). Together, these results suggest that the mvRNA template is the innate immune agonist, rather than the aberrant RNA products derived from the mvRNA template, and that innate immune activation is dependent on a sequence-specific interaction between the active IAV RNA polymerase and the mvRNA template.

### T-loops affect viral polymerase activity and IFN-β promoter activation

**Fig. 2.**
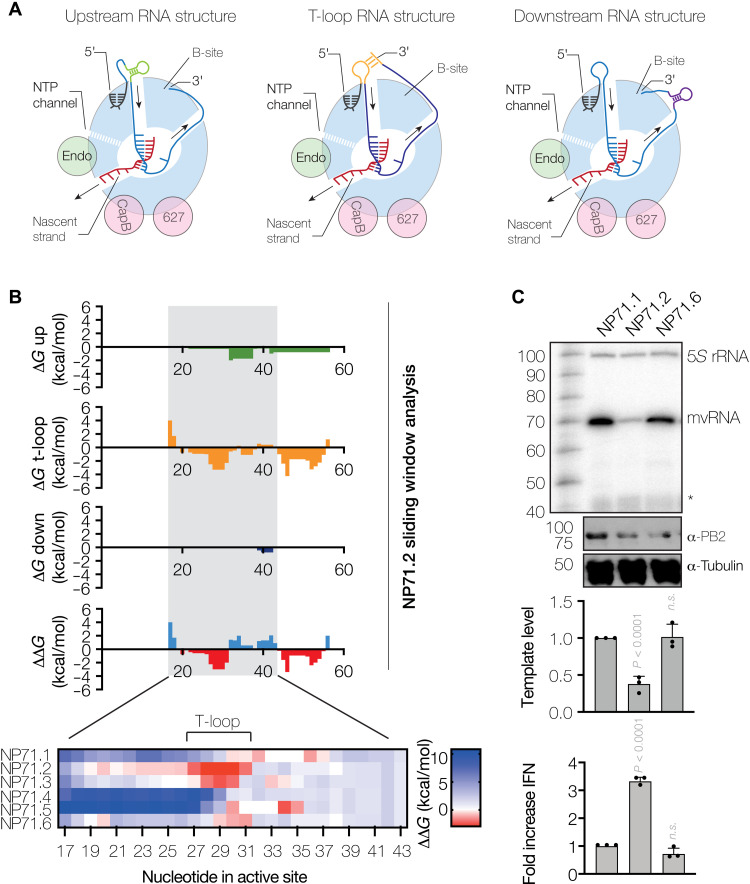
T-loops induce RNA polymerase stalling. (**A**) Schematic of RNA structure formation upstream, around (t-loop), and downstream of the RNA polymerase. (**B**) Δ*G* values for RNA structures forming upstream (green), around (t-loop, orange), or downstream (purple) of the RNA polymerase were computed using a sliding window approach. The difference in Δ*G* (ΔΔ*G*) between the formation of a t-loop and structures forming upstream or downstream the RNA polymerase was computed and shown in the bottom graph. Heatmap shows zoom in on ΔΔ*G* values computed for middle of the mvRNA templates used. (**C**) Replication of the NP71.1, NP71.2, or the destabilized NP71.6 mvRNA templates in HEK293T cells by the WSN RNA polymerase. RNA levels were analyzed by primer extension, and the ability of mvRNA replication to induce IFN promoter activity was analyzed using a luciferase reporter assay. Nonspecific primer extension signals in (C) are indicated with *. Data from three biological repeats are shown.

The vRNA template enters and leaves the active site of the IAV RNA polymerase as a single strand through the entry and exit channels, respectively (Fig. 2A) (*30*–*32*). However, the IAV genome contains various RNA structures that need to be unwound (*33*). Moreover, unwinding of these structures may lead to the formation of transient RNA structures upstream or downstream of the RNA polymerase that may modulate RNA polymerase activity (Fig. 2A), while base pairing between a part of the template that is entering the RNA polymerase and a part of the template that has just been duplicated may trap the RNA polymerase in a template loop (t-loop) (Fig. 2A). To systematically analyze what (transient) RNA structures are present during replication, we used a sliding window algorithm to calculate the minimum free energy (Δ*G*) for every putative t-loop and every putative secondary RNA structure upstream and downstream of the RNA polymerase (fig. S6, A and B). For each position analyzed, we excluded 20 nt from the folding analysis for the footprint of the IAV RNA polymerase (*30*) and 12 nt from the 5′ terminus, which is stably bound by the RNA polymerase before replication termination. As shown in Fig. 2B and fig. S6C, our analysis revealed that NP71.2 and NP71.3 are unique among the engineered mvRNA templates in forming t-loop structures around nucleotide 29 of the positive-sense replicative intermediate (cRNA) but not the negative sense (fig. S6D), suggesting that t-loops in the positive-sense mvRNA template modulate RNA polymerase activity. The likelihood that the t-loops form in the context of other secondary structures were calculated as the difference (ΔΔ*G*) between the computed Δ*G* values for the individual structures (Fig. 2B).

To confirm that t-loops affect RNA polymerase processivity and IFN-β promoter activity, we replaced two A-U base pairs of the NP71.2 t-loop duplex with two G-U base pairs, creating NP71.6 (Fig. 2B and fig. S7A). Using our sliding window analysis, we confirmed that this mutation would make t-loop formation near nucleotide 29 less favorable (Fig. 2B). Following the expression of NP71.1, NP71.2, and NP71.6, we found that replication of the NP71.6 mvRNA was increased relative to the NP71.2 mvRNA, our control mvRNAs, and the NP71.1 mvRNA (Fig. 2C and fig. S7B). In addition, destabilization of the t-loop reduced the induction of the IFN-β promoter activity (Fig. 2C). By contrast, when we mutated the stem of the t-loop of the NP71.2 mvRNA template such that the t-loop around nucleotide 29 was maintained (fig. S7C; NP71.7 and NP71.8), replication remained reduced and IFN-β reporter activity increased relative to the NP71.1 mvRNA (fig. S7, C and D). We also observed again that replication of mvRNAs with a t-loop led to the production of short aberrant RNA products that likely contained internal deletions. However, increases in aberrant RNA levels were not correlated with increases in IFN-β reporter activity, in line with the results in Fig. 1C and fig. S1, and indicated that the mvRNA template is the agonist of IFN-β reporter activity. Analysis of our control mvRNA templates showed that NP56 and NP76 contain weak t-loops in the first half of both the positive- and negative-sense template, while stronger t-loops exist in the second half for the NP56 template (fig. S6E). Together, these results indicate that t-loops can negatively affect IAV RNA synthesis and stimulate innate immune signaling during IAV replication.

### T-loops reduce RNA polymerase processivity in vitro

**Fig. 3.**
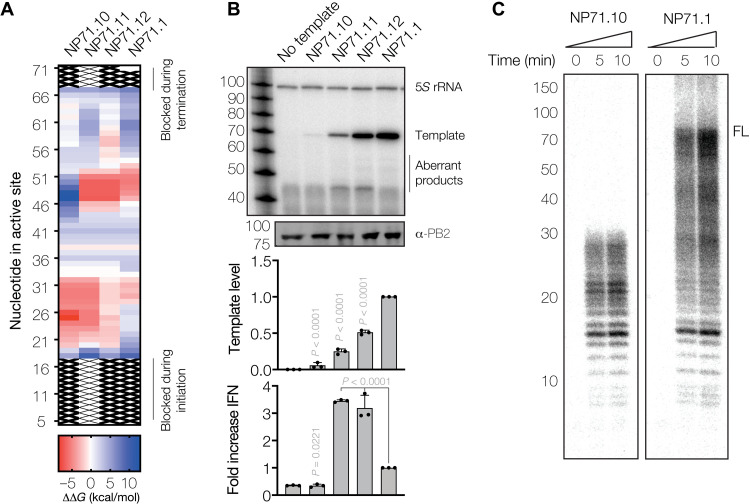
mvRNA t-loops stall RNA polymerase activity and induce IFN-β promoter activation. (**A**) Heatmap showing ΔΔ*G* as estimate for the likelihood of t-loop formation. (**B**) Replication of mvRNA templates. Top panel shows primer extension analysis. Graphs show quantification of template RNA level and IFN-β promoter activation. Data from three biological repeats are shown. rRNA, ribosomal RNA. (**C**) Analysis of IAV RNA polymerase activity in vitro.

The mvRNAs NP71.2 and NP71.3 contain a t-loop in the first half of the positive-sense mvRNA template. To confirm that t-loops also affect RNA polymerase activity in the negative sense, we engineered three additional 71-nt-long mvRNA templates with t-loops in different locations of the template (NP71.10 to NP71.12) (Fig. 3A and table S1). Expression of these mvRNA templates together with the subunits of the vRNA polymerase in HEK293T cells led to strongly reduced NP71.10 and NP71.11 mvRNA levels and slightly reduced NP71.12 mvRNA levels (Fig. 3B). In line with our other results (Figs. 1C and 2C), IFN-β promoter activity was increased for the NP71.11 and NP71.12 templates relative to the NP71.1 mvRNA, while the NP71.10 mvRNA did not induce IFN-β promoter activity, likely because it was too poorly or not fully replicated (Fig. 3B).

To investigate whether t-loops affect RNA polymerase processivity in vitro, we purified the WSN RNA polymerase from HEK293T cells using tandem-affinity purification (TAP) (*34*) and incubated the enzyme with the NP71.1 and NP71.10 mvRNA templates in the presence of NTPs. Following denaturing polyacrylamide gel electrophoresis (PAGE) and autoradiography, we observed a main product of approximately 71 nt in reactions containing the NP71.1 control mvRNA (Fig. 3C). By contrast, incubations with the NP71.10 mvRNA template resulted in products up to approximately 27 nt in length, in agreement with the location of the t-loop in the first half of the mvRNA template (Fig. 3A). Moreover, the observed partial extension of the product offered a possible explanation for the reduced RNA levels in cell culture and the lack of IFN-β promoter activity induction by the NP71.10 template (Fig. 3B).

### T-loops do not induce template release in vitro

To investigate whether t-loop containing templates remained stably bound to the RNA polymerase or triggered template dissociation, we immobilized mOrange-tagged RNA polymerase on magnetic RFP-trap beads and incubated these immobilized complexes with radiolabeled template. After removal of unbound template by three washes with binding buffer, adenosine-guanine dinucleotide (ApG) and nucleoside triphosphates (NTP) were added to initiate RNA synthesis and complexes incubated at 30°C for 15 min. Next, the immobilized complexes were washed three times to remove dissociated RNA, and the reactions stopped with formaldehyde/EDTA loading dye. Analysis of the bound and unbound radiolabeled RNA levels by dot blot and autoradiography showed no difference among the NP71.1, NP71.10, and NP71.11 templates (fig. S8A). To rule out that released template was rebound upon dissociation from the RNA polymerase, we added excess unlabeled NP71.1 template as RNA polymerase trap at the start of the reaction. Again, no difference between the templates was observed (fig. S8A).

To confirm that the immobilized RNA polymerases were active, we immobilized RNA polymerase bound to unlabeled template mvRNA on magnetic beads as described above. Next, we added ApG, NTPs, and radiolabeled guanosine 5′-triphosphate and incubated the immobilized complexes at 30°C for 15 min. Following the removal of unincorporated NTPs by three washes with binding buffer, the nascent RNA in solution and associated with the immobilized complexes was analyzed by denaturing PAGE and autoradiography. As shown in fig. S8B, partially extended and full-length nascent RNAs remained associated with the immobilized RNA polymerases. Partially extended nascent RNAs were also found in the unbound fraction. Addition of inactive RNA polymerase (PB1a) to serve as encapsidating polymerase in RNA polymerase dimers increased the release of partially extended nascent RNAs but not the release of full-length RNAs. Together, these results suggest that t-loops do not induce template release upon RNA polymerase stalling and that partially extended nascent strands can be separated by the RNA polymerase from the template strand and released.

### PB1 K669A increases t-loop sensitivity and IFN-β promoter activation

**Fig. 4.**
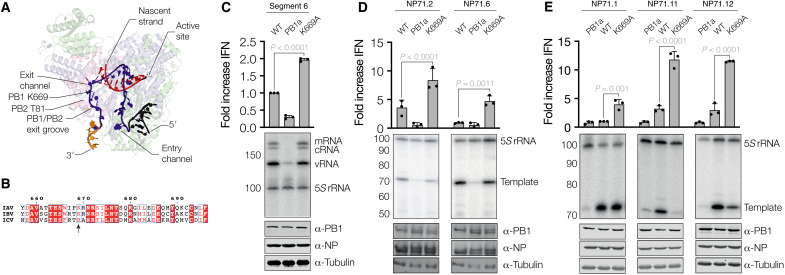
Mutation near template exit channel increases RNA polymerase sensitivity to t-loops. (**A**) Structure of the pretermination complex of the bat IVA RNA polymerase (PDB 6SZU). The 5′ and 3′ ends of the template are shown in black and gold, respectively. The body of the template is shown in dark blue, and the nascent RNA is shown in red. Location of PB1 K669 and PB2 T81 is indicated. (**B**) Amino acid alignment of PB1 C terminus. PB1 K669 is indicated with an arrow. (**C**) IFN-β promoter activity and segment 6 vRNA levels in the presence of WT and K669A RNA polymerases. For the segment 6 vRNA template, the mRNA, cRNA, and vRNA species are indicated. 5*S* rRNA and Western blot for PB1 subunit expression are shown as loading control. (**D** and **E**) IFN-β promoter activity and mvRNA template levels for five NP71 mvRNA templates in the presence of WT and K669A RNA polymerases. RNA levels were analyzed by primer extension. 5*S* rRNA and Western blot for PB1 subunit, NP, and tubulin expression are shown as loading control. Data from three biological repeats are shown.

In the IAV RNA polymerase elongation complex, the 3′ terminus of the template is guided out of the template exit channel via an exit groove on the outside of the thumb subdomain. This groove consists of PB1 and PB2 residues and leads to promoter binding site B (Figs. 2A and 4A) (*15*, *31*). Because this exit groove and the template entry channel reside next to each other at the top of the RNA polymerase (Figs. 2A and 4A), perturbation of the path of the 3′ terminus out of the exit channel may stabilize t-loop formation, reduce RNA polymerase activity, and increase IFN-β promoter activation (Figs. 2A and 4A). In line with this hypothesis, we previously observed that avian adaptive mutations of highly pathogenic IAV RNA polymerases that increase IFN promoter activation in vitro, such as PB2 M81T (Fig. 4A), reside next to the template exit groove (*9*). It is therefore tempting to hypothesize that other mutations near the template exit channel may make the IAV RNA polymerase more sensitive to t-loops.

To test whether dysregulation of the exit groove leads to more IFN-β promoter activation, we mutated PB1 lysine-669, which resides at the start of the exit groove (Fig. 3B), to alanine (K669A). Mutation of this residue had no effect on RNA polymerase activity in the presence of a full-length segment 6 template (Fig. 3C) (*35*) or the NP71.1 and NP71.6 mvRNA templates that do not contain a stable t-loop (Fig. 3, D and E). However, when we expressed the K669A mutant together with the NP71.2, NP71.11, or NP71.12 mvRNAs, which do contain a t-loop in either the positive or negative sense, the K669A mutant displayed greatly reduced RNA polymerase activity (Fig. 3, D and E), suggesting that the K669A mutation increases the processivity defect induced by t-loops. In contrast, the effect of K669A on IFN-β promoter activity was more difficult to interpret because while we observed that the IFN-β promoter activity was considerably increased on the t-loop containing templates (Fig. 3, B to E), the K669A mutant also induced significantly higher IFN-β promoter activity relative to the wild-type (WT) RNA polymerase on the control templates. These results imply that the K669A mutation has two effects: increase the base-level potential of the RNA polymerase to trigger IFN-β promoter activity on templates without a known or destabilized t-loop through an unknown mechanism and make the RNA polymerase more sensitive to disruption by a t-loop and trigger additional IFN-β promoter activity through this mechanism.

### Differential IFN-β promoter activation by natural mvRNAs

**Fig. 5.**
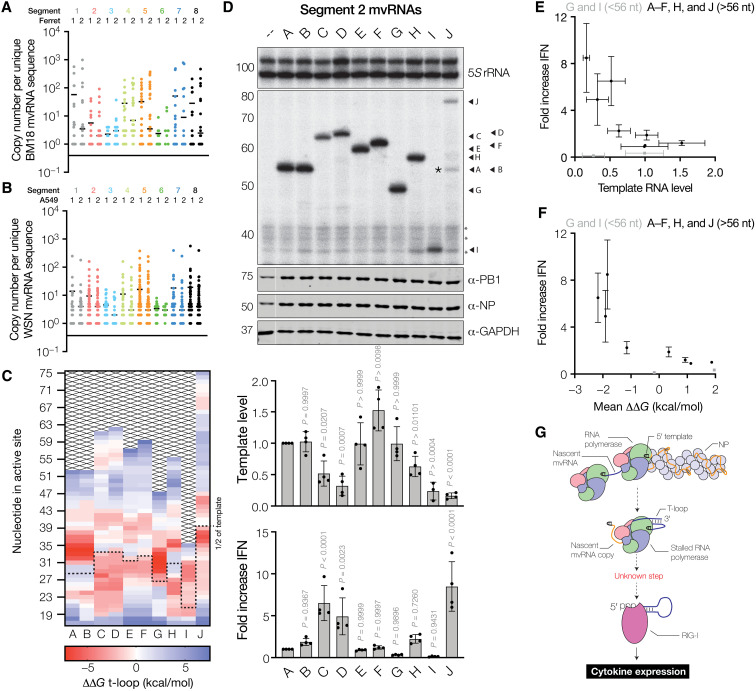
Reduced mvRNA replication in viral infections is correlated with IFN-β promoter activation. (**A**) Number of mvRNA copies per unique mvRNA sequence detected in ferret lungs infected with BM18 or (**B**) A549 cells infected with WSN. In each graph, two biological repeats are shown. (**C**) ΔΔ*G* heatmap of negative-sense template of WSN segment 2–derived mvRNAs. Half of the mvRNA template is indicated with a dotted line. (**D**) Replication of segment 2–derived mvRNAs identified by NGS in HEK293T cells by the WSN RNA polymerase. RNA levels were analyzed by primer extension. The ability of mvRNA replication to induce IFN-β promoter activity was analyzed using a luciferase reporter assay. Asterisk (*) indicates nonspecific radioactive signal. Data from four biological repeats are shown. GAPDH, glyceraldehyde-3-phosphate dehydrogenase. (**E**) IFN-β induction is negatively correlated with template replication level. PB1-mvRNA G and I were excluded from the fit to the exponential decay, because they are shorter than the IFN-β promoter induction cut-off of 56 nt. (**F**) IFN-β induction is negatively correlated with ΔΔ*G* of first half of template mvRNA. (**G**) Schematic of aberrant RNA synthesis by the IAV RNA polymerase. T-loops present in some mvRNAs lead to reduced RNA polymerase processivity. This may induce template release and/or binding of the mvRNA template to RIG-I. Host factor Acidic Nuclear Phosphoprotein 32 Family Member A (ANP32A), which plays a key role during cRNA and vRNA synthesis, is not shown for clarity (*16*, *43*).

mvRNAs are produced during IAV infection in vitro and in vivo (*9*). To study how their sequence and abundance varies, we analyzed RNA extracted from ferret lungs 1 day after infection with BM18 for 1 day and A549 cells infected with WSN for 8 hours (see files S1 to S4 for mvRNA sequences). Although no quantitative comparisons can be made because of the different infection conditions, we do find a notably similar variation in mvRNA sequence and abundance (Fig. 5, A and B).

To investigate the implications of these mvRNA differences on the activation of the IFN-β promoter, we cloned 10 WSN segment 2 mvRNAs (randomly selected over a range of copy numbers and lengths; fig. S9A) into pPolI plasmids (mvRNAs A to J; table S2). Analysis of the ΔΔ*G* values for these mvRNAs revealed potential t-loops in the first half of the sequence for mvRNAs C, D, H, and J and potential t-loops in the second half of the sequence for mvRNAs E, F, and G (Fig. 5C). Subsequent expression of the authentic WSN mvRNAs alongside the WSN RNA polymerase in HEK293T cells showed significant differences in mvRNA amplification (Fig. 5D). These differences were correlated with the abundance detected by next-generation sequencing (NGS) for seven of the cloned mvRNAs (fig. S9B). In addition, we observed that replication of mvRNAs C, D, and J leads to the appearance of products shorter than the template mvRNA (Fig. 5D) and that the appearance of these products is correlated with a reduced replication of the template mvRNA, in line with our findings in Fig. 1.

To investigate whether the different segment 2 mvRNA levels influenced the innate immune response, we measured the IFN-β promoter activity. We found that IFN-β promoter activity varied greatly, with mvRNAs C, D, and J inducing the strongest response (Fig. 5D). Templates I and G, the two shortest mvRNAs at 52 and 40 nt long, induced the lowest IFN-β promoter activity, in line with our previous observations that short mvRNAs <56 nt do not stimulate RIG-I and Fig. 1C. With mvRNAs I and G excluded because of their short size, these observations indicate that the IFN-β promoter activity is negatively correlated with mvRNA template level for mvRNAs >56 nt (Fig. 5E). Moreover, in line with our hypothesis presented in Fig. 1 that t-loops in the first half of the template affect RNA polymerase processivity, the IFN-β promoter activity was negatively correlated with the mean ΔΔ*G* of the first half of the template (Fig. 5F). Weaker correlations were observed between the mvRNA length and IFN-β induction or the mvRNA length and mvRNA replication (fig. S10, A and B).

To exclude that a differential recognition of the mvRNAs was responsible for the observed anticorrelation, we isolated the total RNA from HEK293T transfections and retransfected equal amounts of these RNA extracts into HEK293T cells. As shown in fig. S11A, we observed no significant difference among the segment 2 mvRNAs longer than 56 nt. The mvRNAs G and I failed to induce a strong response because of their short length. To exclude that the different mvRNA levels had been the result of their different effects on the innate immune response, we also expressed the segment 2 mvRNAs in *MAVS*^−/−^ IFN::luc HEK293 cells (*29*). Following expression of the segment 2 mvRNAs, we observed no IFN-β promoter activity (fig. S11B). Primer extension analysis showed no significant reduction in mvRNA steady-state levels compared to WT cells (fig. S11C), indicating that the replication of authentic mvRNAs is not affected by innate immune activation.

To confirm that mvRNAs from other viral segments have differential effects on the innate immune response, we cloned two segment 3 mvRNAs and four segment 4 mvRNAs (table S3) from the mvRNA sequences identified during infection into pPol expression plasmids and transfected these plasmids into HEK293T cells. As shown in fig. S12, PA and hemagglutinin (HA) mvRNAs induced both high and low levels of IFN-β promoter activity compared with our NP71.1 control. Together, these results indicate that viral infections produce mvRNAs with different potentials to induce IFN-β promoter activity and that t-loops play a key role in the potential of mvRNAs to induce IFN-β promoter activity by affecting the ability of the RNA polymerase to efficiently replicate them.

## DISCUSSION

Two factors important for inducing an innate immune response in IAV infections are active viral replication and the binding of vRNA molecules to RIG-I (*14*). We here studied the effect of IAV mvRNAs, which do not need viral NP to be replicated by the vRNA polymerase (*26*). We provide evidence that impeded vRNA polymerase processivity by t-loops is a mechanism that contributes to the activation of innate immune signaling by mvRNAs. While we have no direct assay to measure or visualize t-loop formation in mvRNAs yet and can thus not rule out other or additional mechanisms, we propose that t-loops form when the 3′ terminus or a sequence near the 3′ terminus of the template can hybridize with an upstream part of the template (Fig. 2A). We suspect that the RNA polymerase can unwind a single t-loop, but the formation of several successive t-loops in the first half of the mvRNA stalls the RNA polymerase (Fig. 3C). It is presently still unclear why a strong correlation between reduced processivity and t-loops is observed with t-loops in the first half of the template and not with downstream t-loops.

It is unclear how RIG-I gains access to the t-loop containing mvRNA once the polymerase has stalled (Fig. 4G). We observe that RNA polymerase stalling does not result in release of the RNA template from the active site (Fig. 3D), likely because the RNA polymerase remains associated with the 5′ terminus of the template before replication termination. This means that it is still unclear how mvRNA templates accumulate in the cytoplasm and mitochondria (fig. S2). It is possible that nuclear RIG-I or another host factor directly interacts with the stalled RNA polymerase complex or the t-loop, and we hope to address this question in the future.

It is possible that t-loops affect influenza RNA polymerase activity on full-length vRNAs or DVGs. However, it is more likely that t-loops form only on partially formed RNPs or NP-less templates, because NP may modulate the presence and location of secondary RNA structures. During vRNA synthesis, NP dissociates and binds vRNA in a manner that is coordinated by the vRNA polymerase. When NP levels are reduced, aberrant RNPs or NP-less RNA products may form in which secondary RNA structures that are absent in the presence of NP contribute to t-loop formation and RNA polymerase stalling (*33*). This model could explain how reduced viral NP levels stimulate aberrant RNA synthesis and innate immune activation (*9*, *36*, *37*). We also observe that a mutation near the template exit channel increases the RNA polymerase sensitivity to t-loops (Fig. 4). We previously observed that avian adaptive mutations, such as PB2 N9D or M81T, reside near the template exit channel of highly pathogenic IAV RNA polymerases and that they stimulate IFN-β promoter activity (*9*). It is thus tempting to speculate that these mutations make the RNA polymerase more sensitive to mvRNAs, which are produced at high levels by highly pathogenic IAV RNA polymerases, and that this sensitivity leads to increased RNA polymerase stalling by t-loops and IFN-β promoter activation.

During viral infection, mvRNA molecules of various lengths and abundancies are produced (*9*). We find that, in contrast to our previous assumption (*9*), mvRNA abundance may not be the best estimate for innate immune activation. We therefore propose an updated model in which mvRNAs that are poorly replicated contribute most to the activation of the innate immune system and thus that activation of the innate immune response is dependent on a template sequence context, as observed for paramyxoviruses (*28*). Although we cannot rule out that additional mechanisms contribute to sequence-specific differences in innate immune activation among vRNA templates, our findings open up additional avenues for research as it is possible that potent viral innate immune agonists share the ability to reduce the processivity of the RNA polymerase on vRNA molecules.

## MATERIALS AND METHODS

### Viral protein and RNA expression plasmids

pcDNA3-based plasmids expressing influenza A/WSN/33 (H1N1) proteins PB1, PB2, PA, NP, PB2-TAP, and the active site mutant PB1a (D445/D446A) have been described previously (*9*, *38*, *39*). Mutation K669A was introduced into the pcDNA3-PB1 plasmid by site-directed mutagenesis. mvRNA templates were expressed under the control of the cellular RNA polymerase I promoter from pPolI plasmids. PB1 mvRNA templates were generated by site-directed mutagenesis PCR deletion of pPolI-PB1. Short vRNA templates were created on the basis of the pPolI-NP47 plasmid using the Spe I restriction site.

### Luciferase assay plasmids

Firefly luciferase reporter plasmid under the control of the *IFNB* promoter [pIFΔ(−116)lucter] and constitutively expressing *Renilla* luciferase plasmid (pcDNA3-*Renilla*) were described previously (*9*). The *MAVS*-FLAG expression vector and corresponding empty vector were cloned on the basis of the pFS420, using the *MAVS* WT plasmid (pEF-HA-*MAVS*) (*40*).

### Cells, transfections, and infections

HEK293T, Madin-Darby canine kidney, and A549 cells were originally sourced from the American Type Culture Collection. All cells were routinely screened for mycoplasma. HEK293 WT and *MAVS*^−/−^ cells expressing luciferase under the control of the *IFNB* promoter were a gift from J. Rehwinkel and were described previously (*29*). All cell cultures were grown in Dulbecco’s modified Eagle medium (Sigma-Aldrich) with 10% fetal bovine serum (Sigma-Aldrich) and 1% l-glutamine (Sigma-Aldrich). Transfections of HEK293T or HEK293 cell suspensions were performed using Lipofectamine 2000 (Invitrogen) and Opti-MEM (Invitrogen) following the manufacturer’s instructions, and transfection of confluent, adherent HEK293T cells were performed using polyethylenimine (PEI) (Sigma-Aldrich) and Opti-MEM. Infections were performed at MOI 3 as described previously (*9*).

### Antibodies and Western blotting

IAV proteins were detected using rabbit polyclonal antibodies anti-PB1 (GTX125923, GeneTex), anti-PB2 (GTX125926, GeneTex), and anti-NP (GTX125989, GeneTex) diluted 1:1000 in TBSTM [tris-buffered saline (TBS)/0.1% Tween 20 (Sigma-Aldrich)/5% milk]. Cellular proteins were detected using the rabbit polyclonal antibodies anti–glyceraldehyde-3-phosphate dehydrogenase (GTX100118, GeneTex) diluted 1:4000 in TBSTM and anti-RNA Pol II (ab5131, Abcam) diluted 1:100 in TBSTM; the mouse monoclonal antibodies anti-MAVS E-3 (sc-166583, Santa Cruz Biotechnology) diluted 1:200 in TMSTM and MitoTracker [113-1] (ab92824, Abcam) diluted 1:1000 TBSTM; and the rat monoclonal antibody anti-tubulin (MCA77G, Bio-Rad) diluted 1:1000 in TBSTM. Mouse monoclonal antibody anti-FLAG M2 (F3165, Sigma-Aldrich) diluted at 1:2000 was used to detect MAVS-FLAG. Secondary antibodies IRDye 800 donkey anti-rabbit (926-32213, LI-COR), IRDye 800 goat anti-mouse (926-32210, LI-COR), IRDye 680 goat anti-mouse (926-68020, LI-COR), and IRDye 680 goat anti-rat (926-68076, LI-COR) were used to detect Western signals with a LI-COR Odyssey scanner.

### RNP reconstitution assays and RNA sequence analysis

Infections and RNA analyses using primer extensions were performed as described previously (*9*, *41*). mvRNA identification from NGS data was essentially performed, as described previously (*9*), using data deposited in the Sequence Read Archive under accession number SUB3758924. Aberrant RNA products observed in various experiments were gel-extracted, Topo-cloned, and sequenced using Sanger sequencing. Alignments were analyzed using Clustal Omega and visualized using Espript 3. T-loop analysis was performed using a custom Python script. Briefly, 20 nt of the template sequence were blocked off to represent the footprint of the vRNA polymerase. This footprint was then moved in 1-nt increments along the template (fig. S6, A and B). T-loop formation was assessed by computing the Δ*G* of duplex formation between a stretch of 10-nt upstream of the footprint and 10-nt downstream of the footprint. The formation of upstream and downstream structures was computed for 24-nt windows (the footprint of NP) upstream and downstream of the moving footprint. The ΔΔ*G* was computed by subtracting The ViennaRNA package commands duplexfold and cofold were used to compute the Δ*G* values (*42*).

### Luciferase-based IFN expression assays

To measure IFN expression in RNP reconstituted HEK293T or HEK293 cells, luciferase assays were carried out 24 hours after transfection. RNP reconstitutions were carried out in a 24-well format by transfecting 0.25 μg of the plasmids pcDNA3-PB1, pcDNA3-PB2, pcDNA3-PA, pcDNA3-NP, and a pPolI plasmid expressing a mvRNA template. HEK293T and HEK293 cells were additionally cotransfected with 100 ng of the plasmid pIFΔ(−116)lucter and 10 ng of the plasmid pcDNA3-*Renilla*. Cells were harvested in phosphate-buffered saline (PBS) and resuspended in an equal volume of Dual-Glo reagent (Promega), followed by Dual-Glo Stop & Glo reagent (Promega). Firefly and *Renilla* luminescence were measured after 10-min incubation with each reagent respectively as per the manufacturer’s instructions for the Dual-Glo Luciferase Assay System (E2920, Promega) using the GloMax luminometer (Promega).

### IAV polymerase purification and in vitro activity assay

Influenza virus A/WSN/33 (H1N1) recombinant polymerases were purified from HEK293T cells. Ten-centimeter plates of adherent cells were transfected with 3 μg of pcDNA3-PB1, pcDNA3-PB2-TAP, and pcDNA3-PA with PEI (Sigma-Aldrich). Forty-eight hours after transfection, cells were harvested in PBS and lysed on ice for 10 min in 500 μl of lysis buffer [50 mM Hepes (pH 8.0), 200 mM NaCl, 25% glycerol (Sigma-Aldrich), 0.5% Igepal CA-630 (Sigma-Aldrich), 1 mM β-mercaptoethanol (Bio-Rad), 1× phenylmethylsulfonyl fluoride (PMSF) (Sigma-Aldrich), and 1× protease inhibitor cocktail tablet (Roche)]. Lysates were cleared by centrifugation at 17,000*g* for 5 min at 4°C, diluted in 2 ml of NaCl (Sigma-Aldrich), and bound to prewashed immunoglobulin G Sepharose beads (Cytiva) for 2 hours at 4°C. Beads were prewashed 3× in binding buffer [10 mM Hepes (pH 8.0), 0.15 M NaCl, 0.1% Igepal CA-630, 10% glycerol, and 1× PMSF]. After binding, beads were washed 3× in binding buffer and 1× in cleavage buffer [10 mM Hepes (pH 8.0) (Sigma-Aldrich), 0.15 M NaCl, 0.1% Igepal CA-630, 10% glycerol, 1× PMSF, and 1 mM dithiothreitol]. Beads were cleaved with AcTEV protease (Invitrogen) overnight at 4°C and cleared by centrifugation at 1000*g* for 1 min as described previously (*41*). Activity assays using immobilized RNA polymerase were performed using an RNA polymerase with an mOrange-tag on the PB2 subunit. The purified polymerase was immobilized using magnetic RFP-trap beads (ChromoTek).

### Cell fractionation

Fractionation of transfected cells into cytoplasmic, mitochondrial, and nuclear components was carried out using the Abcam Cell Fractionation Kit (Abcam) following the manufacturer’s instructions, with volumes adjusted on the basis of the number of cells. Samples of unfractionated whole cells in buffer A were retained as input controls. Whole cells and subcellular fractions were dissolved in TRIzol for RNA extraction and analyzed as described above or in 10% SDS protein-loading buffer for protein expression analysis by SDS-PAGE and Western blot.

### Statistical testing

Statistical testing was carried out using GraphPad Prism 9 software. Error bars represent SDs, and either individual data or group mean values are plotted. One-way analysis of variance (ANOVA) with Dunnett’s test for multiple comparisons was used to compare multiple-group means to a normalized mean (e.g., IFN induction or RNA template replication). Two-way ANOVA with Sidak’s test for multiple comparisons was used to compare multiple pairs of group means (e.g., between two cell types, HEK293 WT to HEK293 *MAVS*^−/−^).

## Supplementary Materials

### This PDF file includes:

Figs. S1 to S12 Tables S1 to S3 Files S1 to S4 Supplemental Script

## Notes

View/request a protocol for this paper from *Bio-protocol*.
