# Supplementary material for: Transient RNA structures cause aberrant influenza virus replication and innate immune activation
Source: Sci Adv. 2022 Sep 9;8(36):eabp8655. doi: 10.1126/sciadv.abp8655 (PMC9462681; doi:10.1126/sciadv.abp8655)
Supplement: Supplementary file 1 — Figs. S1 to S12 Tables S1 to S3 Files S1 to S4 Supplemental Script [file sciadv.abp8655_sm.pdf]

Science Advances
AAAS

Supplementary Materials for

Transient RNA structures cause aberrant influenza virus replication and innate immune activation

Hollie French *et al.*

Corresponding author: Aartjan J.W. te Velthuis, aj.te.velthuis@princeton.edu

Sci. Adv. **8**, eabp8655 (2022)
DOI: 10.1126/sciadv.abp8655

This PDF file includes:

Figs. S1 to S12
Tables S1 to S3
Files S1 to S4
Supplemental Script

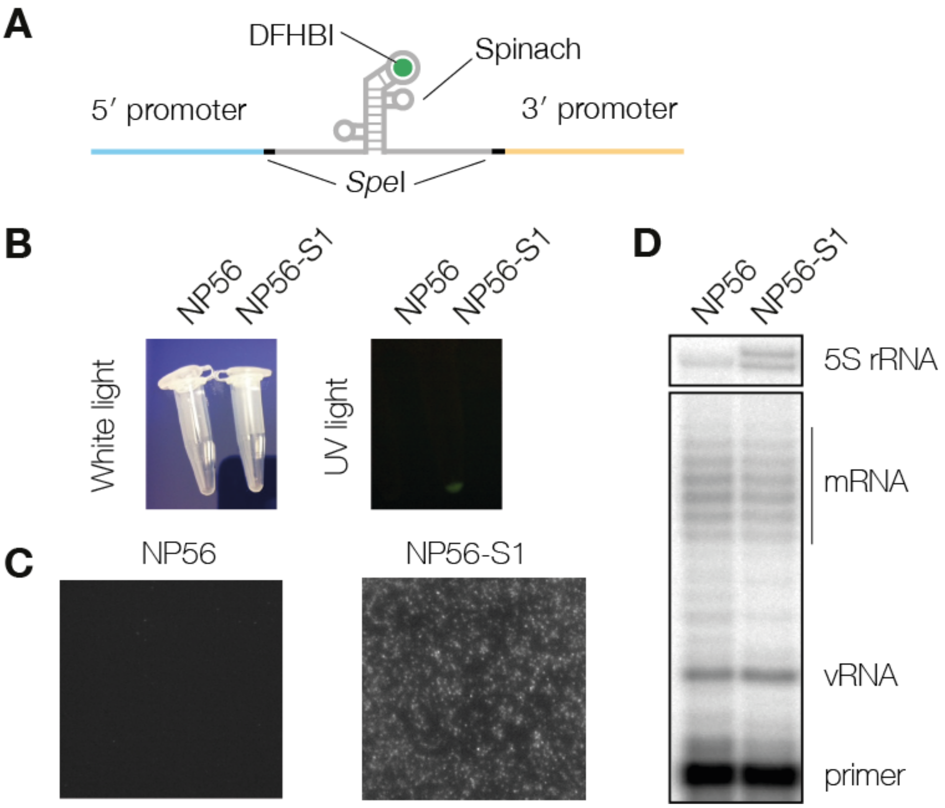

Figure S1. Effect of a single spinach aptamer on mvRNA replication. A) To rule out that a single RNA structure upstream of the RNA polymerase reduces activity, we engineered the NP56 mvRNA template and inserted the RNA aptamer Spinach, creating NP56-S1. B) A Spinach-containing mvRNAs generated in vitro demonstrated fluorescence after addition of DFHBI relative to the 56-nt control mvRNA, confirming that the aptamer folds properly in the context of the IAV promoter sequence. C) TIRF microscopy image of a 56-nt mvRNA or Spinach-containing mvRNA generated in vitro in the presence of DFHBI. D) After transfection of plasmids encoding the Spinach-containing mvRNA NP56-S1 into HEK293T cells and analysis of the RNA produced using primer extension, no difference in replication was observed relative to our NP56 control mvRNA.

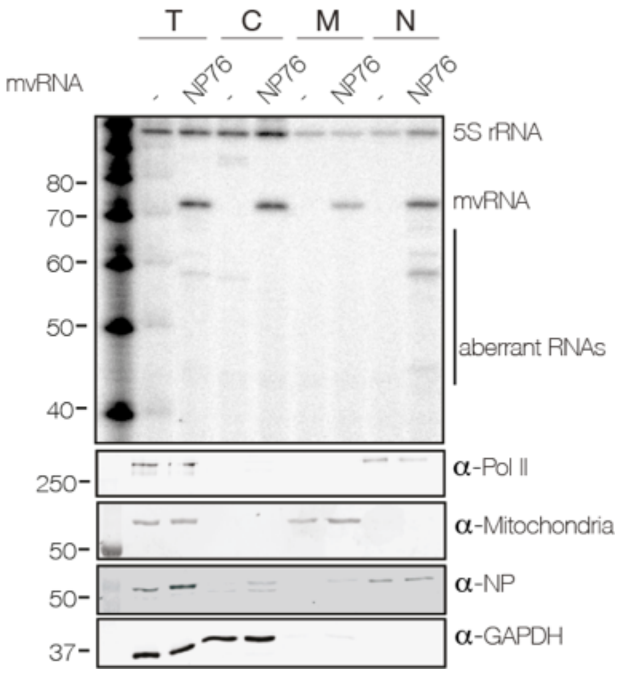

Figure S2. Fractionation of HEK 293T cells transfected with IAV RNA polymerase and a 76 nt-long segment 5-derived mvRNA. Top panel shows primer extension analysis of RNA extracted from fractions. Bottom four panels show western blot analysis. For each lane, comparable amounts were loaded.

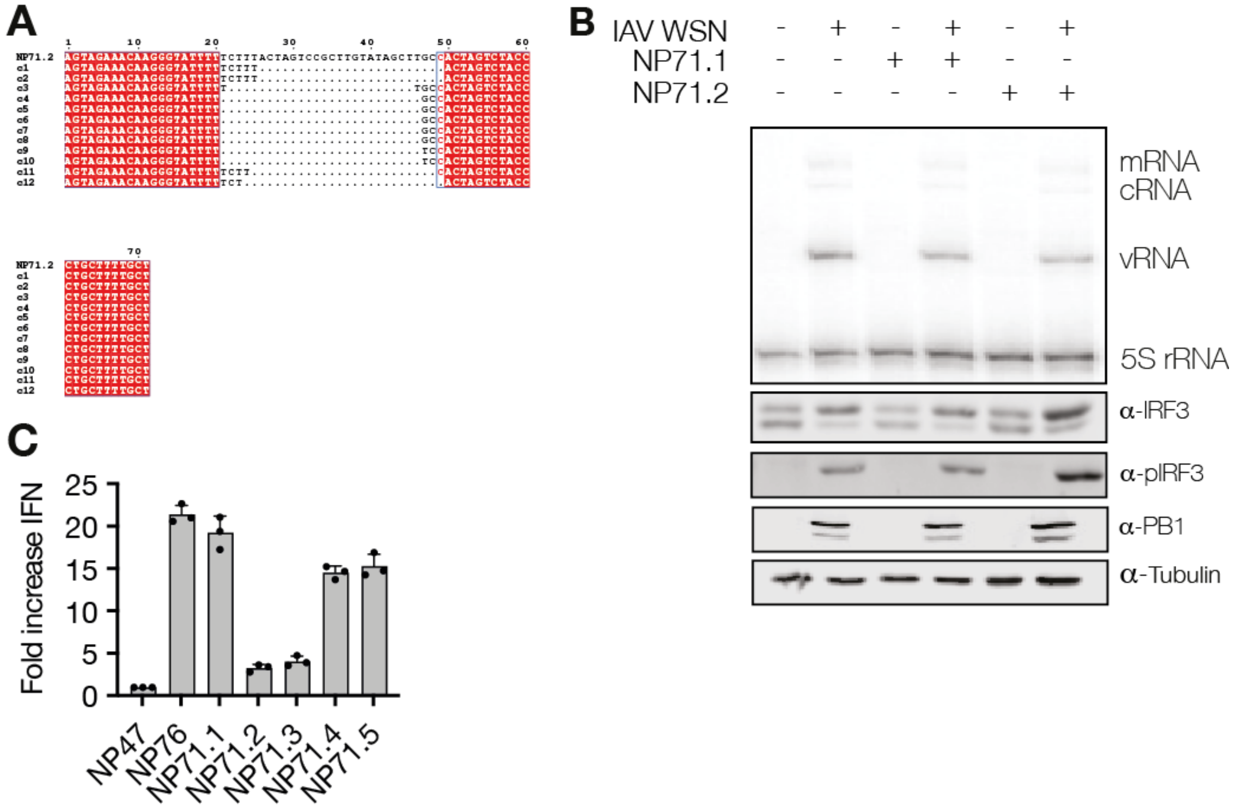

Figure S3. Aberrant RNA sequences, effect of exogenous mvRNAs on infection, and retransfection of total RNA. A) Alignment of aberrant RNA products isolated from in vitro replication assays. B) IFN-β promoter activity induced by the retransfection of total RNA isolated from HEK 293T cells transfected with plasmids expressing segment 5-derived mvRNAs and the viral RNA polymerase subunits. Luciferase signal was normalized to the NP47 mvRNA, which does not trigger innate immune responses. E) Induction of IRF3 phosphorylation by pre-transfected, exogenous mvRNAs during infection with IAV A/WSN/33 (H1N1). mvRNA amplification could not be detected by primer extension, likely because exogenous templates poorly compete with endogenous viral RNAs for replication.

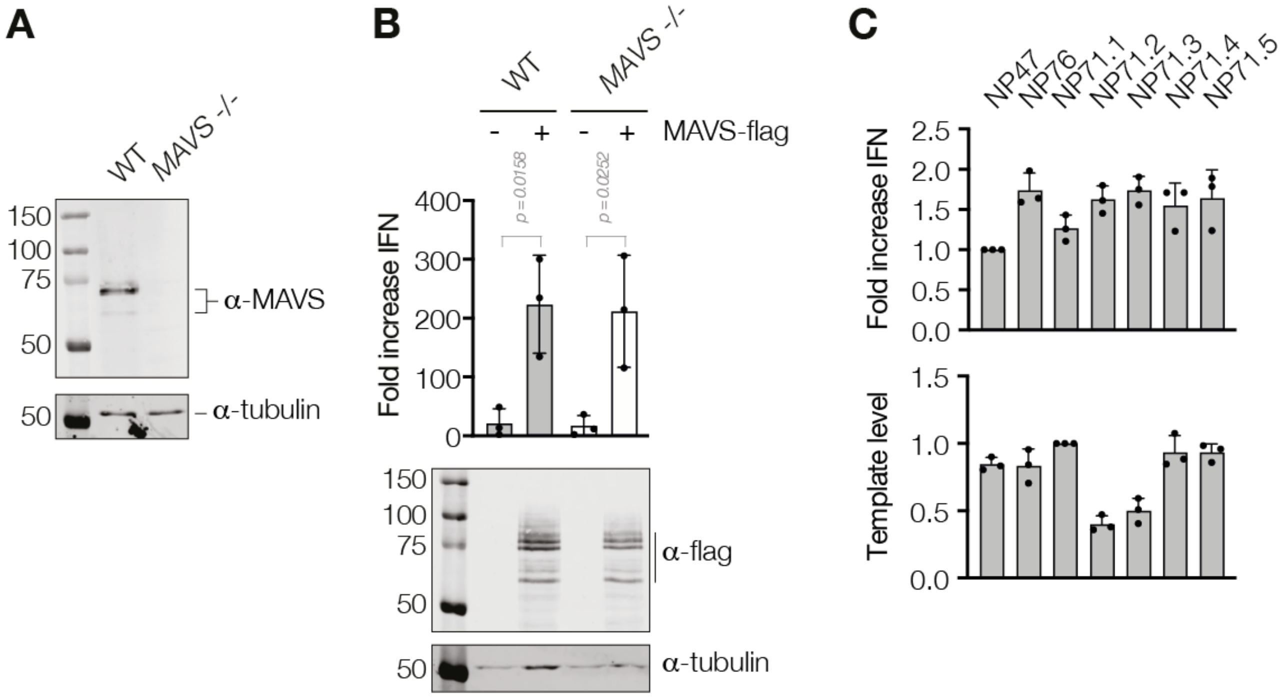

Figure S4. Characterization of *MAVS*$^{-/-}$ HEK 293 cells and mvRNA replication in *MAVS*$^{-/-}$ HEK 293 cells. A) Western blot analysis of MAVS expression in wildtype or *MAVS*$^{-/-}$ HEK 293 cells. B) IFN-β promoter activity in wildtype or *MAVS*$^{-/-}$ HEK 293 cells following transfection of a plasmid expressing MAVS-flag. Bottom panel shows western blot of MAVS-flag expression. C) IFN-β promoter activity of HEK 293 *MAVS*$^{-/-}$ cells expressing mvRNAs NP71.1-NP71.5. and primer extension analysis of mvRNA NP71.1-NP71.5 replication in HEK 293 *MAVS*$^{-/-}$ cells. P-values were computed using student t-test.

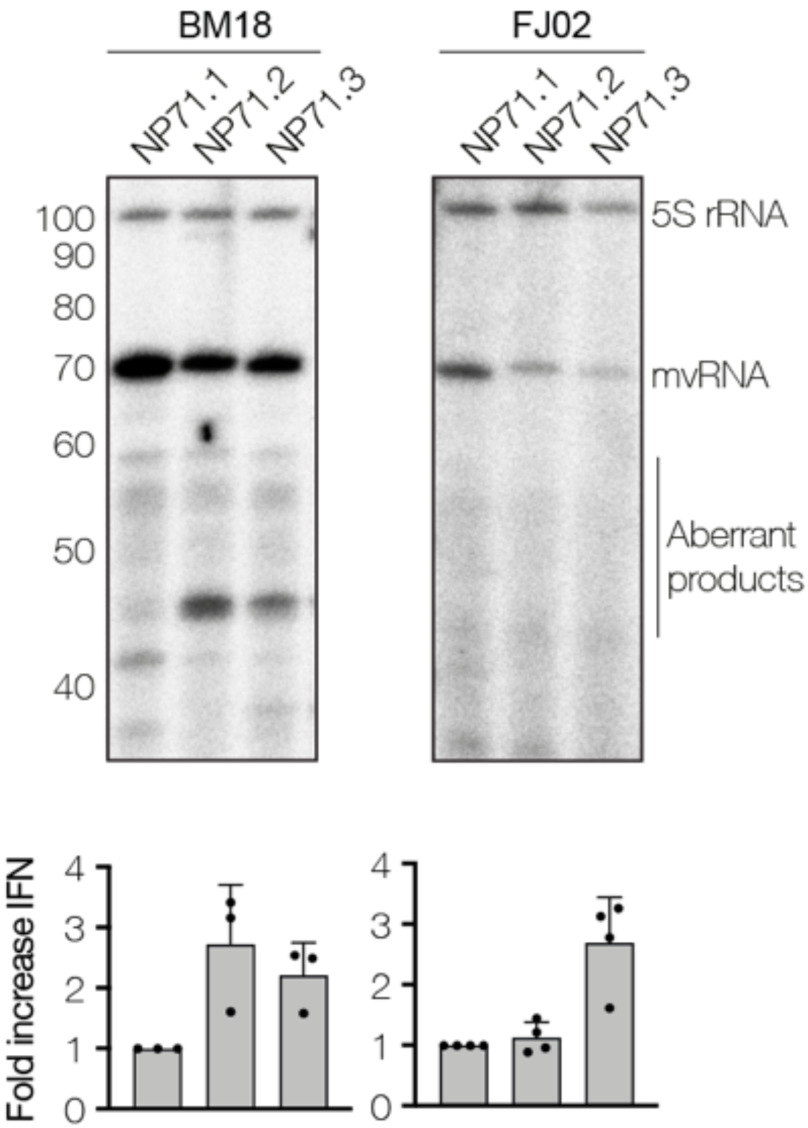

Figure S5. Replication of model mvRNAs in HEK 293T cells by the IAV A/Brevig Mission/1/18 (BM18) or A/duck/Fujian/01/02 (FJ02) RNA polymerases. The ability of these reactions to induce IFN-β promoter activity was analyzed using a luciferase reporter assay.

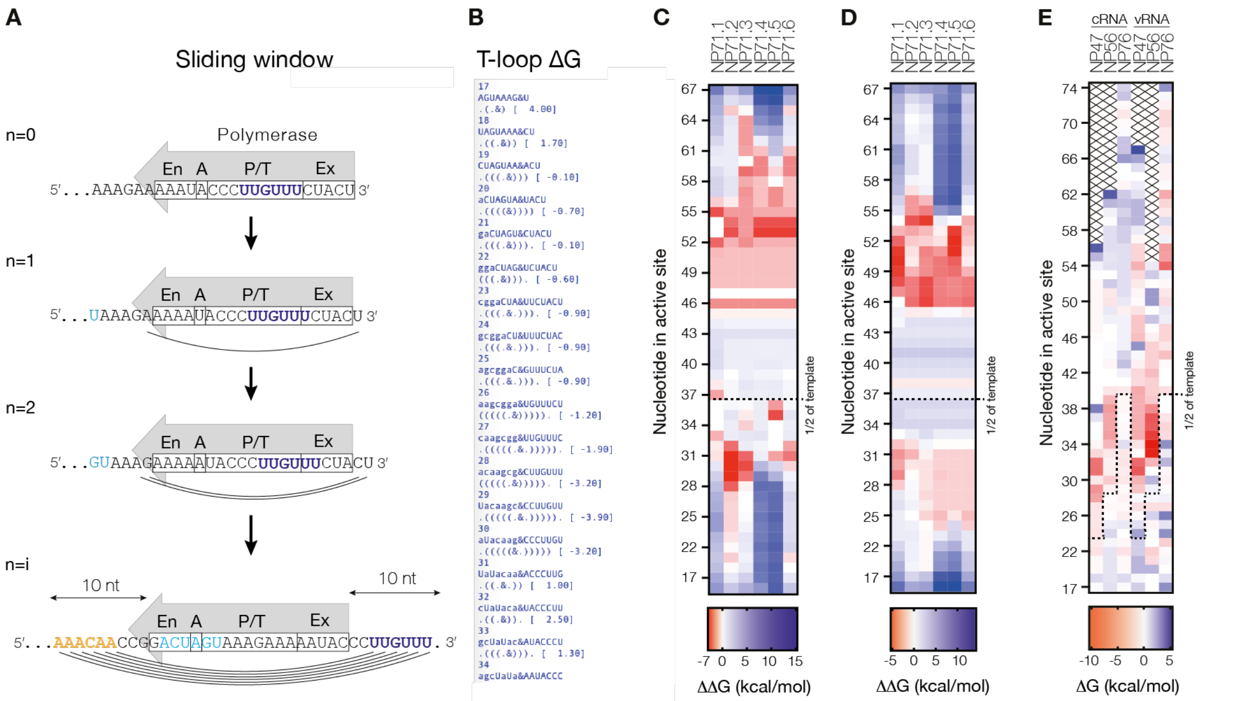

Figure S6. Sliding-window analysis of the free energy (ΔG) of transient secondary RNA structure formation near the influenza A virus RNA polymerase. A) To calculate the ΔG for a t-loop, 10 nt on either side of the polymerase were allowed to fold using the duplex-fold algorithm of the ViennaRNA package. To calculate the ΔG for alternative structures, cofold of the ViennaRNA package was used. The ΔΔG for the likelihood of t-loop formation was calculated by subtracting the upstream and downstream ΔG values from the t-loop ΔG value. B) Example of Python script output. C) ΔΔG values for positive sense 71-nt long mvRNA templates. D) ΔΔG values for negative sense 71-nt long mvRNA templates. E) t-loop ΔG values for positive sense and negative sense NP47, NP56 and NP76 mvRNA templates.

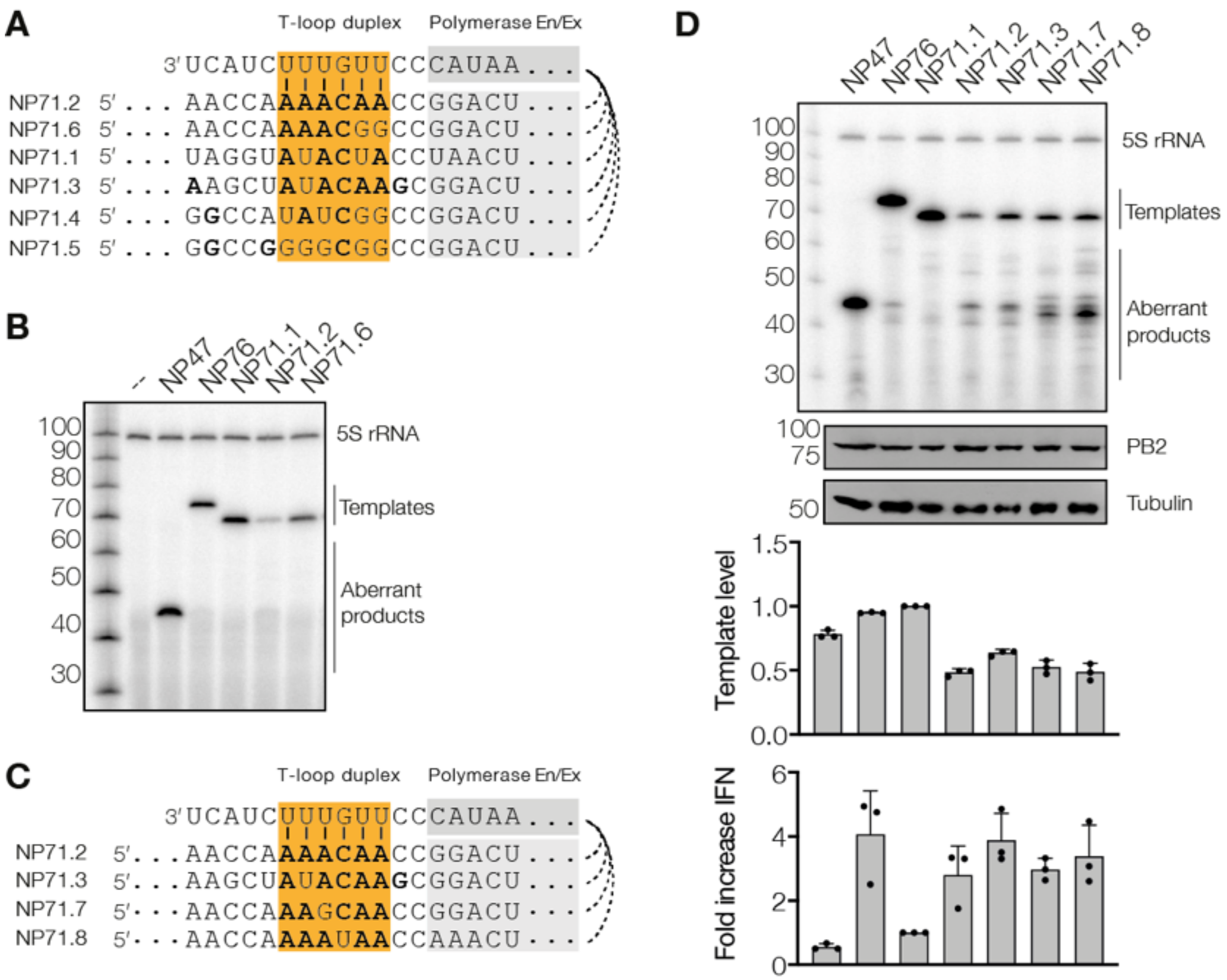

Figure S7. Replication of engineered mvRNAs and their ability to induce IFN-β promoter activity. A) Alignment of t-loops (shaded orange) in NP71 mvRNA templates. Location of RNA polymerase entry (En) and exit (Ex) channel is shaded gray. B) Primer extension analysis of RNA extracted from HEK 293T cells expressing NP71 mvRNA templates. C) Alignment of t-loops (shaded orange) of additional NP71 mvRNA templates. Location of RNA polymerase entry and exit channel is shaded gray. D) Primer extension analysis of RNA extracted from HEK 293T cells expressing NP71 mvRNA templates. Second and third panel show western blot analysis. Top graph shows quantification of mvRNA template level, while bottom graph shows IFN-β promoter activity analysis using a luciferase reporter.

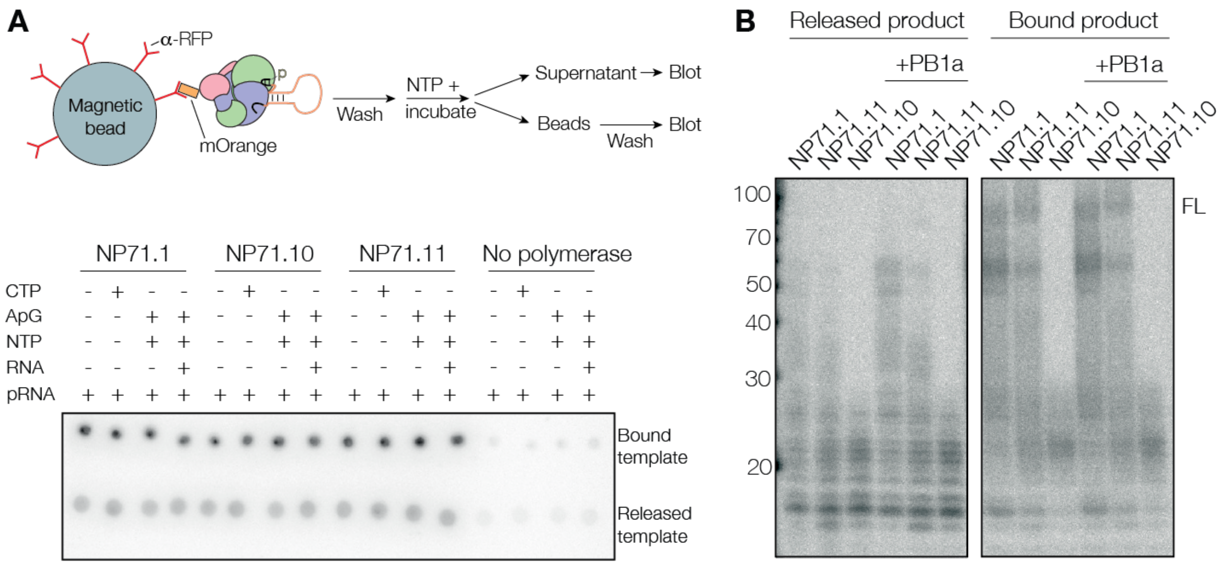

Figure S8. Analysis of IAV RNA polymerase template binding and release on different mvRNA templates in vitro. A) Binding of radiolabeled RNA templates by mOrange-tagged, immobilized IAV RNA polymerase in the absence and presence of ApG and NTPs. B) Binding of radiolabeled RNA products by mOrange-tagged, immobilized IAV RNA polymerase in the absence and presence of free inactive IAV RNA polymerase (PB1a).

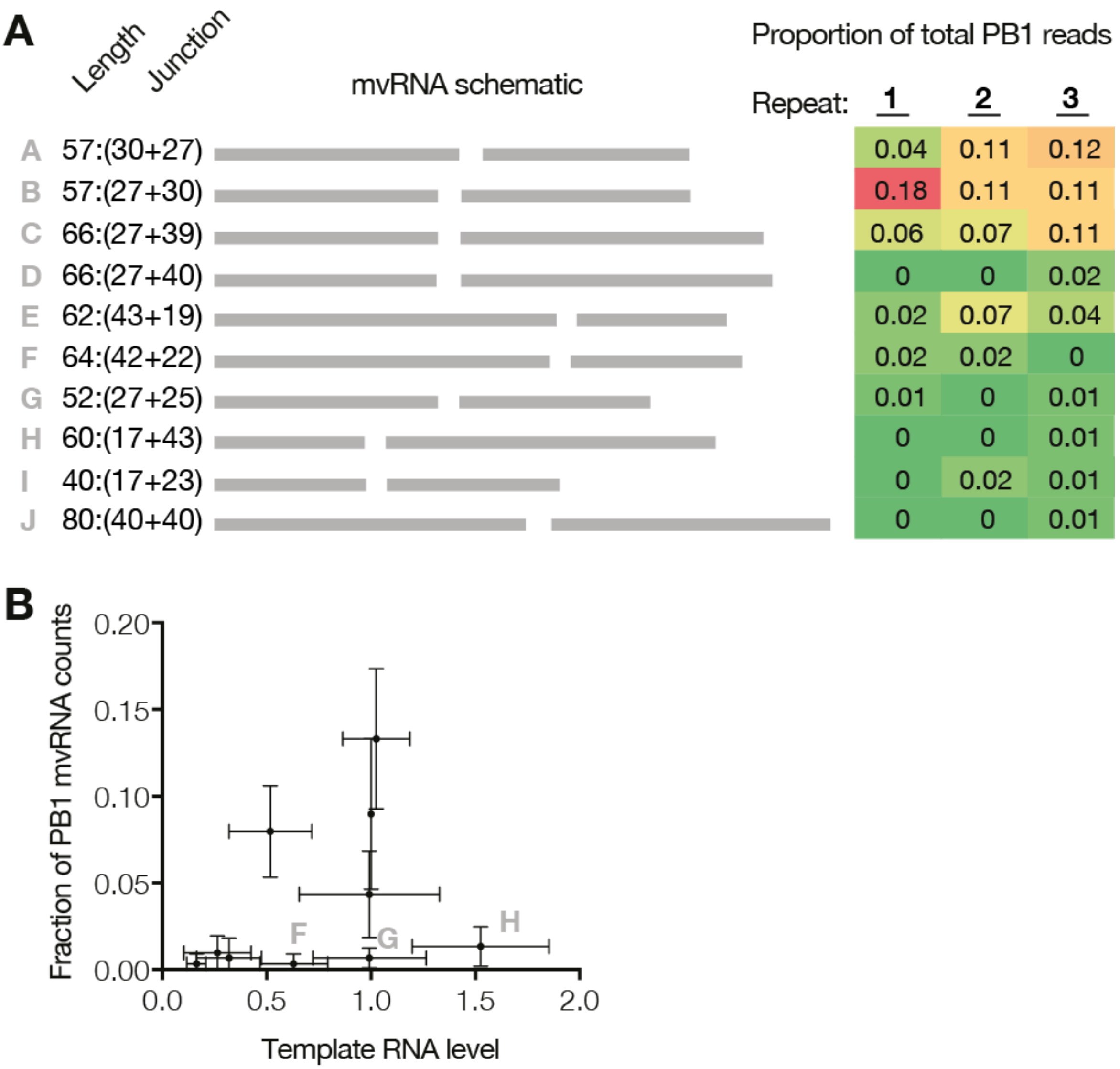

Figure S9. Selection of segment 2 mvRNAs. A) Segment 2 mvRNA sizes and abundance in three next generation sequencing experiments. B) Relation between mvRNA level in transfection assay and mvRNA read counts in next generation sequencing. The mvRNAs F, G and H are indicated.

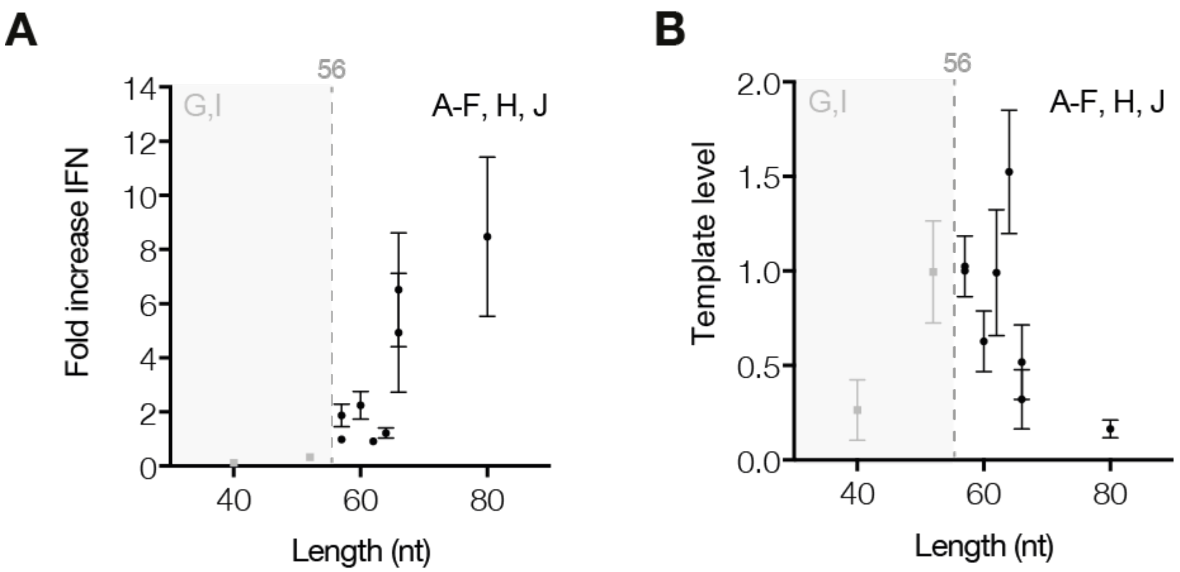

Figure S10. Relation between the A) mvRNA template length and the IFN-β promoter activity induction, or B) the mvRNA template length and mvRNA replication.

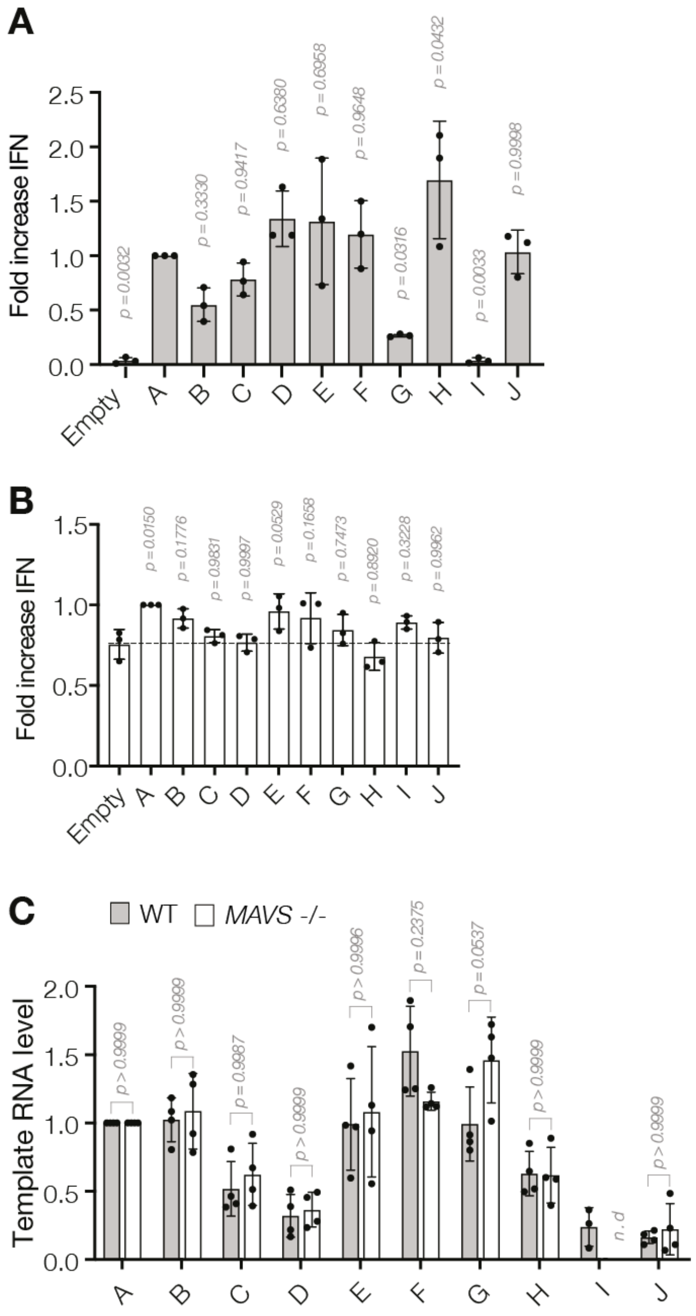

Figure S11. Segment 2 mvRNA replication in *MAVS*$^{-/-}$ HEK 293 cells. A) IFN-β promoter activity measured after retransfection of total RNA extracted from HEK 293T cells expressing segment 2 mvRNAs into HEK 293T cells. B) IFN-β promoter activity induced by expression of segment 2 mvRNAs in *MAVS*$^{-/-}$ HEK 293 cells. C) Primer extension analysis of segment 2 mvRNAs levels in wildtype and *MAVS*$^{-/-}$ HEK 293 cells. P-values were computed using one-way ANOVA with multiple-corrections relative to template A or the empty plasmid control.

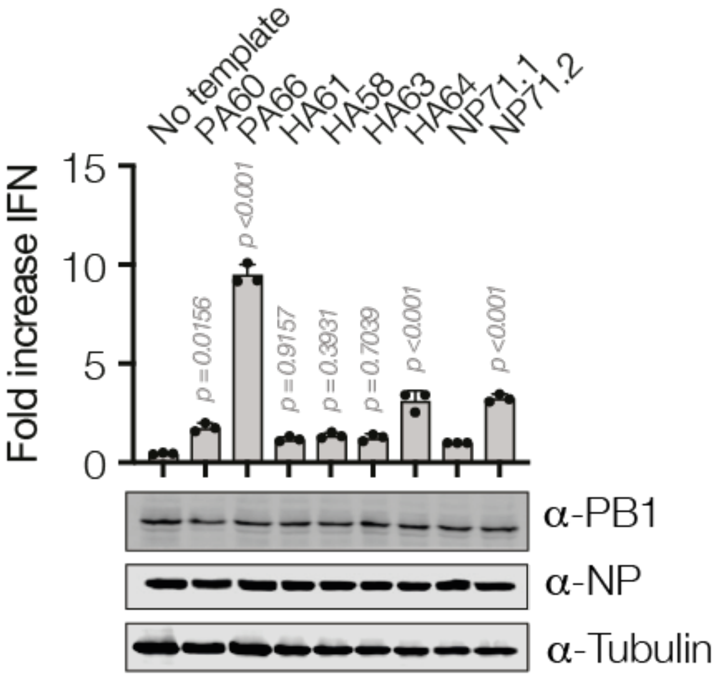

Figure S12. Induction of IFN beta promoter activity by segment 3 and 4 mvRNAs. P-values were computed using one-way ANOVA with multiple-corrections relative to the NP71.1 mvRNA template.

Table S1. Sequences of mvRNA templates used.

**Name**	**Internal lab reference**	**Sequence 5' and 3' (vRNA)**
NP71.1	GC33	AGUAGAAACAAGGGUAUUUUUCUUUACUAGUUAGGUAGUAUA CCUAGUAACUAGUCUACCCUGCUUUUGCU
NP71.2	GC50.1	AGUAGAAACAAGGGUAUUUUUCUUUACUAGUCCGGUUGUUU UGGUUGCCACUAGUCUACCCUGCUUUUGCU
NP71.3	GC50.2	AGUAGAAACAAGGGUAUUUUUCUUUACUAGUCCGCUUGUAU AGCUUGCCACUAGUCUACCCUGCUUUUGCU
NP71.4	GC67	AGUAGAAACAAGGGUAUUUUUCUUUACUAGUCCGGCCGAUA UGGCCGCCACUAGUCUACCCUGCUUUUGCU
NP71.5	GC83	AGUAGAAACAAGGGUAUUUUUCUUUACUAGUCCGGCCGCCC CGGCCGCCACUAGUCUACCCUGCUUUUGCU
NP71.6	GC50.9	AGUAGAAACAAGGGUAUUUUUCUUUACUAGUCCGGCCGUUU UGGUUGCCACUAGUCUACCCUGCUUUUGCU
NP71.7	GC50.3	AGUAGAAACAAGGGUAUUUUUCUUUACUAGUCCGGUUCUUU UGGUUGCCACUAGUCUACCCUGCUUUUGCU
NP71.8	GC50.4	AGUAGAAACAAGGGUAUUUUUCUUUACUAGUCCGGUUGCUU UGGUUGCCACUAGUCUACCCUGCUUUUGCU
NP47	NP47	AGUAGAAACAAGGGUAUUUUUCUUUACUAGUCUACCCUGCU UUUGCU
NP56	NP56	AGUAGAAACAAGGGUAUUUUUCUUUCUCGAGCGUACUAGUC UACCCUGCUUUUGCU
NP76	NP76	AGUAGAAACAAGGGUAUUUUUCUUUACUAGUGAUUUCGAUG UCACUCUGUGAGUGAUUAUCUACCCUGCUUUUGCU
NP71.10	GC50_13	AGUAGAAACAAGGGUAUUUUUCUUUACUAGUGGCAGCAAAAG CAGGGUAACUAGUCUACCCUGCUUUUGCU
NP71.11	GC50_15	AGUAGAAACAAGGGUAUUUUUCUUUACUAGUGGCAGCAAAAG CACCCAUACUAGUCUACCCUGCUUUUGCU
NP71.12	GC50_16	AGUAGAAACAAGGGUAUUUUUCUUUACUAGUGGCUCUAAAAG CACCCAUACUAGUCUACCCUGCUUUUGCU

Table S2. Cloned PB1 WSN mvRNAs.

Name	Length (nt)	Sequence 5' and 3' (vRNA)
PB1 A	57	AGUAGAAACAAGGCAUUUUUUCAUGAAAUCCAUUCAAAUGGU UUGCCUGCUUUCGCU
PB1 B	57	AGUAGAAACAAGGCAUUUUUUCAUGAAGGACAUUCAAAUGGU UUGCCUGCUUUCGCU
PB1 C	66	AGUAGAAACAAGGCAUUUUUUCAUGAAGGACAAGCUAAACAU UCAAAUGGUUUGCCUGCUUUCGCU
PB1 D	67	AGUAGAAACAAGGCAUUUUUUCAUGAAGGACAAGCUAAAUCA UUCAAAUGGUUUGCCUGCUUUCGCU
PB1 E	62	AGUAGAAACAAGGCAUUUUAAGUCGGAUUGACAUCCAUUCAA AUGGUUUGCCUGCUUUCGCU
PB1 F	64	AGUAGAAACAAGGCAUUUUUUCAGUCGGAUUGACAUCCAUUC AAAUGGUUUGCCUGCUUUCGCU
PB1 G	52	AGUAGAAACAAGGCAUUUUUUCAUGCAUUCAAAUGGUUUGCC UGCUUUCGCU
PB1 H	60	AGUAGAAACAAGGCAUUUUUUCAUGAAGGACAAGCUAAAUUC AGUUUGCCUGCUUUCGCU
PB1 I	40	AGUAGAAACAAGGCAUUUUUUCAGUUUGCCUGCUUUCGCU
PB1 J	80	AGUAGAAACAAGGCAUUUUUUCAUGAAGGACAAGCUAAAUUC GGAUUGACAUCCAUUCAAAUGGUUUGCCUGCUUUCGCU

Table S3. Cloned mvRNAs based on t-loop analysis.

Name	Sequence 5' and 3' (vRNA)
PA66	AGUAGAAACAAGGUACUUUUUUGGACAGUAUGGAUAGCACAUUUUGAAUCAGUACCUGCUUUCGCU
PA60	AGUAGAAACAAGGUACUUUUUUGGACAGUAUGCCAUUUUGAAUCAGUACCUGCUUUCGCU
HA61	AGUAGAAACAAGGGUGUUUUUCCUUAUAUUUCUGAAAUCCUAAUCUUCCCCUGCUUUUGCU
HA58	AGUAGAAACAAGGGUGUUUUUCCUUAUAUUUCUGAAAUCCUAUUCCCCUGCUUUUGCU
HA63	AGUAGAAACAAGGGUGUUUUUCCUUAUAUUUCUGAAAUGUUUUUAUUUUCCCCUGCUUUUGCU
HA64	AGUAGAAACAAGGGUGUUUUUCCUUAUAUUUCUGAAAUCCUAAUCUCAUUCCCCUGCUUUUGCU

Supplemental Fasta file 1. WSN mvRNA sequences.
>WSN_NP_61
AGCAAAAGCAGGGTAGATAATCACTCACAGAGTGACATAGAAAAATACCCTTGTTTCTACT
>WSN_PB1_57
AGCGAAAGCAGGCAAACCATTTGAATGGATTTCATGAAAAAATGCCTTGTTTCTACT
>WSN_PB1_57
AGCGAAAGCAGGCAAACCATTTGAATGTCCTTCATGAAAAAATGCCTTGTTTCTACT
>WSN_PB1_66
AGCGAAAGCAGGCAAACCATTTGAATGTTTAGCTTGTCCTTCATGAAAAAATGCCTTGTTTCTACT
>WSN_NP_65
AGCAAAAGCAGGGTAGATAATCACTCACAGAGTGACATCTAAAGAAAAATACCCTTGTTTCTACT
>WSN_HA_44
AGCAAAAGCAGGGGAATATAAGGAAAAACACCCTTGTTTCTACT
>WSN_NA_70
AGCAAAAGCAGGAGTTTAAATGAATCCAAACCTGACAAGTAGTTTGTTCAAAAAACTCCTTGTTTCTACT
>WSN_PB1_62
AGCGAAAGCAGGCAAACCATTTGAATAGCTTGTCCTTCATGAAAAAATGCCTTGTTTCTACT
>WSN_NP_70
AGCAAAAGCAGGGTAGATAATCACTCACAGAGTGACATCGAAATTAAAGAAAAATACCCTTGTTTCTACT
>WSN_PB1_64
AGCGAAAGCAGGCAAACCATTTGAATGGATGTCAATCCGACTTAAAAAATGCCTTGTTTCTACT
>WSN_PB1_69
AGCGAAAGCAGGCAAACCATTTGAATGGATGTTAGCTTGTCCTTCATGAAAAAATGCCTTGTTTCTACT
>WSN_NA_69
AGCAAAAGCAGGAGTTTAAATGAATCCAAACCAGAAAGTAGTTTGTTCAAAAAACTCCTTGTTTCTACT
>WSN_PB1_62
AGCGAAAGCAGGCAAACCATTTGAATGGATGTCAATCCGACTTAAAATGCCTTGTTTCTACT
>WSN_PB1_68
AGCGAAAGCAGGCAAACCATTTGAATGGATGTCAATCCGACTTCATGAAAAAATGCCTTGTTTCTACT
>WSN_HA_60
AGCAAAAGCAGGGGAAATTAGGATTTCAGAAATATAAGGAAAAACACCCTTGTTTCTACT
>WSN_PB1_68
AGCGAAAGCAGGCAAACCATTTGAATGGATGTCAATCCTCCTTCATGAAAAAATGCCTTGTTTCTACT
>WSN_PB1_49
AGCGAAAGCAGGCAAACCATTTGAATTGAAAAAATGCCTTGTTTCTACT
>WSN_PB1_56
AGCGAAAGCAGGCAAACTTTAGCTTGTCCTTCATGAAAAAATGCCTTGTTTCTACT
>WSN_PB2_60
AGCGAAAGCAGGTCAATTATATTCAATATCGAATAGTTTAAAAACGACCTTGTTTCTACT
>WSN_PB1_60
AGCGAAAGCAGGCAAACCATTTGAATGGATGTCAATATGAAAAAATGCCTTGTTTCTACT
>WSN_NP_74
AGCAAAAGCAGGGTAGATAATCACTCACAGAGTGACATCTACGACAATTAAAGAAAAATACCCTTGTTTCTACT
>WSN_NA_73
AGCAAAAGCAGGAGTTTAAATGAATCCAAACCAGAAAACAAGTAGTTTGTTCAAAAAACTCCTTGTTTCTACT
>WSN_NA_62
AGCAAAAGCAGGAGTTTAAATGAATCCAAACTAGTTTGTTCAAAAAACTCCTTGTTTCTACT
>WSN_NA_63
AGCAAAAGCAGGAGTTTAACACCATTGACAAGTAGTTTGTTCAAAAAACTCCTTGTTTCTACT
>WSN_PB2_55
AGCGAAAGCAGGTCAATTATATTCCGAATAGTTTAAAAACGACCTTGTTTCTACT
>WSN_PB1_44
AGCGAAAGCAGGCAAACCATATGAAAAAATGCCTTGTTTCTACT
>WSN_PA_64
AGCGAAAGCAGGTACTGATTCAAATTGCTATCCATACTGTCCAAAAAAGTACCTTGTTTCTACT
>WSN_NP_70
AGCAAAAGCAGGGTAGATAATCACTCACAGAGTGACATCGAAATCATAGAAAAATACCCTTGTTTCTACT
>WSN_HA_46
AGCAAAAGCAGGGGAAAATAAAAAGAAAAACACCCTTGTTTCTACT
>WSN_HA_61
AGCAAAAGCAGGGGAAAATAAAGATTTCAGAAATATAAGGAAAAACACCCTTGTTTCTACT
>WSN_PB1_65
AGCGAAAGCAGGCAAACCATTTGAATGGATGTCAATCCGACTTGAAAAAATGCCTTGTTTCTACT
>WSN_PB1_61
AGCGAAAGCAGGCAAACCATTTGAATGG TTGTCCTTCATGAAAAAATGCCTTGTTTCTACT
>WSN_PB1_67
AGCGAAAGCAGGCAAACCATTTGAATGATTTAGCTTGTCCTTCATGAAAAAATGCCTTGTTTCTACT
>WSN_PB1_60
AGCGAAAGCAGGCAAACCATTTGTAGCTTGTCCTTCATGAAAAAATGCCTTGTTTCTACT
>WSN_PB1_64

AGCGAAAGCAGGCAAACCATGTGAATTTAGCTTGTCCTTCATGAAAAAATGCCTTGTTTCTACT
>WSN_PA_66
AGCGAAAGCAGGTACTGATTTACTATTTGCTATCCATACTGTCCAAAAAAGTACCTTGTTTCTACT
>WSN_NP_69
AGCAAAAGCAGGGTAGATAATCACTCACAGAGTGACATCGAAATCATGGCAAATACCCTTGTTTCTACT
>WSN_NP_70
AGCAAAAGCAGGGTAGATAATCACTCACAGAGTGACATCGAAATCATGGCAAAATACCCTTGTTTCTACT
>WSN_NP_66
AGCAAAAGCAGGGTAGATAATCACTCACAGAGTGACATCTTAAAGAAAAATACCCTTGTTTCTACT
>WSN_NP_64
AGCAAAAGCAGGGTAGATAATCACTCACAGAGTGACATTAAAGAAAAATACCCTTGTTTCTACT
>WSN_NP_57
AGCAAAAGCAGGGTAGATAATCACTCACAGAGTGACAAAAATACCCTTGTTTCTACT
>WSN_NP_59
AGCAAAAGCAGGGTAGATAATCACTCACAGAGTTAAAGAAAAATACCCTTGTTTCTACT
>WSN_NP_55
AGCAAAAGCAGGGTAGATAATCACTCACAGAGAGAAAAATACCCTTGTTTCTACT
>WSN_NA_64
AGCAAAAGCAGGAGTTTAAATGAATCCAAACCATAGTTTGTTCAAAAAACTCCTTGTTTCTACT
>WSN_NA_71
AGCAAAAGCAGGAGTTTAAATGAATCCAACCATTGACAAGTAGTTTGTTCAAAAAACTCCTTGTTTCTACT
>WSN_HA_64
AGCAAAAGCAGGGGAATGAGATTAGGATTTCAGAAATATAAGGAAAAACACCCTTGTTTCTACT
>WSN_PB2_62
AGCGAAAGCAGGTCAATTATATTCAATATGGAGAATAGTTTAAAAACGACCTTGTTTCTACT
>WSN_PB2_57
AGCGAAAGCAGGTCAATTATATTCAATATGTAGTTTAAAAACGACCTTGTTTCTACT
>WSN_PB2_48
AGCGAAAGCAGGTCAATTATATTCATTAAAAACGACCTTGTTTCTACT
>WSN_PB2_54
AGCGAAAGCAGGTCAATTATAGTCGAATAGTTTAAAAACGACCTTGTTTCTACT
>WSN_PB2_49
AGCGAAAGCAGGTCAATTAGAATAGTTTAAAAACGACCTTGTTTCTACT
>WSN_PB2_58
AGCGAAAGCAGGTCAATCAATTAGTGTCGAATAGTTTAAAAACGACCTTGTTTCTACT
>WSN_PB1_68
AGCGAAAGCAGGCAAACCATTTGAATGGATGTCAATCCGACTTTACGAAAAAATGCCTTGTTTCTACT
>WSN_PB1_73
AGCGAAAGCAGGCAAACCATTTGAATGGATGTCAATCCGACTTTCCTTCATGAAAAAATGCCTTGTTTCTACT
>WSN_PB1_61
AGCGAAAGCAGGCAAACCATTTGAATGGATGTCAATCCGACTTAAATGCCTTGTTTCTACT
>WSN_PB1_63
AGCGAAAGCAGGCAAACCATTTGAATGGATGTCAATCCGACTTAAAAATGCCTTGTTTCTACT
>WSN_PB1_80
AGCGAAAGCAGGCAAACCATTTGAATGGATGTCAATCCGAATTTAGCTTGTCCTTCATGAAAAAATGCCTTGTTTCTACT
>WSN_PB1_47
AGCGAAAGCAGGCAAACCATTTGAATGAAAAATGCCTTGTTTCTACT
>WSN_PB1_48
AGCGAAAGCAGGCAAACCATTTGAATGAAAAAATGCCTTGTTTCTACT
>WSN_PB1_64
AGCGAAAGCAGGCAAACCATTTGAATGTAGCTTGTCCTTCATGAAAAAATGCCTTGTTTCTACT
>WSN_PB1_62
AGCGAAAGCAGGCAAACCATTTGATTAGCTTGTCCTTCATGAAAAAATGCCTTGTTTCTACT
>WSN_PB1_43
AGCGAAAGCAGGCAAACCATTTAAAAAATGCCTTGTTTCTACT
>WSN_PB1_40
AGCGAAAGCAGGCAAACTGAAAAAATGCCTTGTTTCTACT
>WSN_PB1_50
AGCGAAAGCAGGCAAACTTGTCCTTCATGAAAAAATGCCTTGTTTCTACT
>WSN_NS_47
AGCAAAAGCAGGGTGACAAAGAAATAAAAAACACCCTTGTTTCTACT
>WSN_NP_72
AGCAAAAGCAGGGTAGATAATCACTCACAGAGTGACATCGAAATCATGGAGAAAAATACCCTTGTTTCTACT
>WSN_NP_88
AGCAAAAGCAGGGTAGATAATCACTCACAGAGTGACATCGAAATCATGGGGAGTACGACAATTAAAGAAAAATACCCTTGTTTC
TACT
>WSN_NP_91
AGCAAAAGCAGGGTAGATAATCACTCACAGAGTGACATCGAAATAATGCAGAGGAGTACGACAATTAAAGAAAAATACCCTTGT
TTCTACT
>WSN_NP_81

AGCAAAAGCAGGGTAGATAATCACTCACAGAGTGACATCGAAAGAGTACGACAATTAAAGAAAAATACCCTTGTTTCTACT
>WSN_NP_62
AGCAAAAGCAGGGTAGATAATCACTCACAGAGTGACATCGGAAAAATACCCTTGTTTCTACT
>WSN_NP_57
AGCAAAAGCAGGGTAGATAATCACTCACAGAGTGACATCAATACCCTTGTTTCTACT
>WSN_NP_58
AGCAAAAGCAGGGTAGATAATCACTCACAGAGTGACATCAAATACCCTTGTTTCTACT
>WSN_NP_81
AGCAAAAGCAGGGTAGATAATCACTCACAGAGTGAATGCAGAGGAGTACGACAATTAAAGAAAAATACCCTTGTTTCTACT
>WSN_NP_62
AGCAAAAGCAGGGTAGATAATCACTCATACGACAATTAAAGAAAAATACCCTTGTTTCTACT
>WSN_NP_49
AGCAAAAGCAGGGTAGAGACAATTAAAGAAAAATACCCTTGTTTCTACT
>WSN_NA_72
AGCAAAAGCAGGAGTTTAAATGAATCCAAACCAGAAAAAAGTAGTTTGTTCAAAAAACTCCTTGTTTCTACT
>WSN_NA_70
AGCAAAAGCAGGAGTTTAAATGAATCCAAACCAGACAAGTAGTTTGTTCAAAAAACTCCTTGTTTCTACT
>WSN_NA_69
AGCAAAAGCAGGAGTTTAAATGAATCCAAATTGACAAGTAGTTTGTTCAAAAAACTCCTTGTTTCTACT
>WSN_NA_45
AGCAAAAGCAGGAGTTTAAATTTCAAAAAACTCCTTGTTTCTACT
>WSN_NA_52
AGCAAAAGCAGGAGTTTAAATTAGTTTGTTCAAAAAACTCCTTGTTTCTACT
>WSN_NA_62
AGCAAAAGCAGGAGTTTAAACCATTGACAAGTAGTTTGTTCAAAAAACTCCTTGTTTCTACT
>WSN_M_70
AGCGAAAGCAGGTAGATATTGAAAGATGAGTCTTCTAACCGAGGTCGTAAAAAACTACCTTGTTTCTACT
>WSN_M_47
AGCGAAAGCAGGTAGATATTGAAAGATAAAACTACCTTGTTTCTACT
>WSN_HA_52
AGCAAAAGCAGGGGAAAATAAAAACAACCAGAAAAACACCCTTGTTTCTACT
>WSN_HA_49
AGCAAAAGCAGGGGAAAATAAAAACAAGAAAAACACCCTTGTTTCTACT
>WSN_HA_48
AGCAAAAGCAGGGGAAAATAAAAACAGAAAAACACCCTTGTTTCTACT
>WSN_HA_63
AGCAAAAGCAGGGGAAAATAAAAACATTTCAGAAATATAAGGAAAAACACCCTTGTTTCTACT
>WSN_HA_67
AGCAAAAGCAGGGGAAAATAAAGATTAGGATTTCAGAAATATAAGGAAAAACACCCTTGTTTCTACT
>WSN_HA_61
AGCAAAAGCAGGGGAAAATTAGGATTTCAGAAATATAAGGAAAAACACCCTTGTTTCTACT
>WSN_HA_58
AGCAAAAGCAGGGGAATAGGATTTCAGAAATATAAGGAAAAACACCCTTGTTTCTACT
>WSN_PB2_57
AGCGAAAGCAGGTCAATTATATTCAATATGGAAAGAAAAAACGACCTTGTTTCTACT
>WSN_PB2_69
AGCGAAAGCAGGTCAATTATATTCAATATGGAAAGAGTCGAATAGTTTAAAAACGACCTTGTTTCTACT
>WSN_PB2_67
AGCGAAAGCAGGTCAATTATATTCAATATGTAGTGTCGAATAGTTTAAAAACGACCTTGTTTCTACT
>WSN_PB2_51
AGCGAAAGCAGGTCAATTATATTCAATATTAAAAACGACCTTGTTTCTACT
>WSN_PB2_65
AGCGAAAGCAGGTCAATTATATTCAATATAGTGTCGAATAGTTTAAAAACGACCTTGTTTCTACT
>WSN_PB2_59
AGCGAAAGCAGGTCAATTATATTCAATACGAATAGTTTAAAAACGACCTTGTTTCTACT
>WSN_PB2_62
AGCGAAAGCAGGTCAATTATATTCAATGTGTCGAATAGTTTAAAAACGACCTTGTTTCTACT
>WSN_PB2_52
AGCGAAAGCAGGTCAATTATATTCATAGTTTAAAAACGACCTTGTTTCTACT
>WSN_PB2_63
AGCGAAAGCAGGTCAATTATATTCATTAGTGTCGAATAGTTTAAAAACGACCTTGTTTCTACT
>WSN_PB2_44
AGCGAAAGCAGGTCAATTATATTAAAAACGACCTTGTTTCTACT
>WSN_PB2_60
AGCGAAAGCAGGTCAATTATATTTAGTGTCGAATAGTTTAAAAACGACCTTGTTTCTACT
>WSN_PB2_46
AGCGAAAGCAGGTCAATAATAGTTTAAAAACGACCTTGTTTCTACT
>WSN_PB2_51
AGCGAAAGCAGGTCAAGTGTCGAATAGTTTAAAAACGACCTTGTTTCTACT
>WSN_PB1_73

AGCGAAAGCAGGCAAACCATTTGAATGGATGTCAATCCGACTTTACTTTTCTAAAAAATGCCTTGTTTCTACT
>WSN_PB1_69
AGCGAAAGCAGGCAAACCATTTGAATGGATGTCAATCCGACTTTACTTTTAAAATGCCTTGTTTCTACT
>WSN_PB1_75
AGCGAAAGCAGGCAAACCATTTGAATGGATGTCAATCCGACTTTACTCTTCATGAAAAAATGCCTTGTTTCTACT
>WSN_PB1_65
AGCGAAAGCAGGCAAACCATTTGAATGGATGTCAATCCGACTTTACAAAATGCCTTGTTTCTACT
>WSN_PB1_64
AGCGAAAGCAGGCAAACCATTTGAATGGATGTCAATCCGACTTTAAAAATGCCTTGTTTCTACT
>WSN_PB1_61
AGCGAAAGCAGGCAAACCATTTGAATGGATGTCAATCCGACTAAAATGCCTTGTTTCTACT
>WSN_PB1_73
AGCGAAAGCAGGCAAACCATTTGAATGGATGTCAATCCGACTGTCCTTCATGAAAAAATGCCTTGTTTCTACT
>WSN_PB1_63
AGCGAAAGCAGGCAAACCATTTGAATGGATGTCAATCCCATGAAAAAATGCCTTGTTTCTACT
>WSN_PB1_60
AGCGAAAGCAGGCAAACCATTTGAATGGATGTCAATCTGAAAAAATGCCTTGTTTCTACT
>WSN_PB1_80
AGCGAAAGCAGGCAAACCATTTGAATGGATGTCAATCTGAATTTAGCTTGTCCTTCATGAAAAAATGCCTTGTTTCTACT
>WSN_PB1_63
AGCGAAAGCAGGCAAACCATTTGAATGGATGTCACCTTCATGAAAAAATGCCTTGTTTCTACT
>WSN_PB1_65
AGCGAAAGCAGGCAAACCATTTGAATGGATGTCAGTCCTTCATGAAAAAATGCCTTGTTTCTACT
>WSN_PB1_58
AGCGAAAGCAGGCAAACCATTTGAATGGATGTCCATGAAAAAATGCCTTGTTTCTACT
>WSN_PB1_52
AGCGAAAGCAGGCAAACCATTTGAATGGATGTAAAAATGCCTTGTTTCTACT
>WSN_PB1_63
AGCGAAAGCAGGCAAACCATTTGAATGGACTTGTCCTTCATGAAAAAATGCCTTGTTTCTACT
>WSN_PB1_59
AGCGAAAGCAGGCAAACCATTTGAATGGGTCCTTCATGAAAAAATGCCTTGTTTCTACT
>WSN_PB1_52
AGCGAAAGCAGGCAAACCATTTGAATGCATGAAAAAATGCCTTGTTTCTACT
>WSN_PB1_48
AGCGAAAGCAGGCAAACCATTTTCATGAAAAAATGCCTTGTTTCTACT
>WSN_PB1_61
AGCGAAAGCAGGCAAACCATTTTTTAGCTTGTCCTTCATGAAAAAATGCCTTGTTTCTACT
>WSN_PB1_57
AGCGAAAGCAGGCAAACCATTAGCTTGTCCTTCATGAAAAAATGCCTTGTTTCTACT
>WSN_PB1_60
AGCGAAAGCAGGCAAACTGAATTTAGCTTGTCCTTCATGAAAAAATGCCTTGTTTCTACT
>WSN_PB1_63
AGCGAAAGCAGGCAAACTAGTGAATTTAGCTTGTCCTTCATGAAAAAATGCCTTGTTTCTACT
>WSN_PB1_56
AGCGAAAGCAGGCAAAATTTAGCTTGTCCTTCATGAAAAAATGCCTTGTTTCTACT
>WSN_PA_63
AGCGAAAGCAGGTACTGATTCAAAATGGAAGATTTTGTTCCAAAAAAGTACCTTGTTTCTACT
>WSN_PA_58
AGCGAAAGCAGGTACTGATTCAAAATGGAAGATTTTAAAAAAGTACCTTGTTTCTACT
>WSN_PA_53
AGCGAAAGCAGGTACTGATTCAAAATGGAAGATTAAAGTACCTTGTTTCTACT
>WSN_PA_63
AGCGAAAGCAGGTACTGATTTATTTGCTATCCATACTGTCCAAAAAAGTACCTTGTTTCTACT
>WSN_PA_44
AGCGAAAGCAGGTACTGAGTCCAAAAAAGTACCTTGTTTCTACT
>WSN_NS_59
AGCAAAAGCAGGGTGACAAAGACATAATGGATCCAAACACTAACACCCTTGTTTCTACT
>WSN_NS_60
AGCAAAAGCAGGGTGACAAAGACATAATGGATCCAAATAAAAAACACCCTTGTTTCTACT
>WSN_NS_52
AGCAAAAGCAGGGTGACAAAGACATAATGTAAAAAACACCCTTGTTTCTACT
>WSN_NS_48
AGCAAAAGCAGGGTGACAAAGACATTAAAAAACACCCTTGTTTCTACT
>WSN_NS_44
AGCAAAAGCAGGGTGACAAAGACAAAAACACCCTTGTTTCTACT
>WSN_NS_46
AGCAAAAGCAGGGTGACAAAGACAAAAAAACACCCTTGTTTCTACT
>WSN_NS_45
AGCAAAAGCAGGGTGACAAAGATAAAAAACACCCTTGTTTCTACT
>WSN_NS_67

```
AGCAAAAGCAGGGTGACAAAGTCTCGTTTCAGCTTATTTAATAATAAAAAACACCCTTGTTTCTACT
>WSN_NS_46
AGCAAAAGCAGGGTGACAAATAATAAAAAACACCCTTGTTTCTACT
>WSN_NS_63
AGCAAAAGCAGGGTGACACTCGTTTCAGCTTATTTAATAATAAAAAACACCCTTGTTTCTACT
>WSN_NP_94
AGCAAAAGCAGGGTAGATAATCACTCACAGAGTGACATCGAAATCATGGCGACCAAAGGCACCAAACGATCTTACAAATACCCT
TGTTTCTACT
>WSN_NP_71
AGCAAAAGCAGGGTAGATAATCACTCACAGAGTGACATCGAAATCATGGCAAAAATACCCTTGTTTCTACT
>WSN_NP_74
AGCAAAAGCAGGGTAGATAATCACTCACAGAGTGACATCGAAATCATGGCAAGAAAAATACCCTTGTTTCTACT
>WSN_NP_76
AGCAAAAGCAGGGTAGATAATCACTCACAGAGTGACATCGAAATCATGGCTAAAGAAAAATACCCTTGTTTCTACT
>WSN_NP_91
AGCAAAAGCAGGGTAGATAATCACTCACAGAGTGACATCGAAATCATGGAGAGGAGTACGACAATTAAAGAAAAATACCCTTGT
TTCTACT
>WSN_NP_64
AGCAAAAGCAGGGTAGATAATCACTCACAGAGTGACATCGAAATAAAATACCCTTGTTTCTACT
>WSN_NP_65
AGCAAAAGCAGGGTAGATAATCACTCACAGAGTGACATCGAAAGAAAAATACCCTTGTTTCTACT
>WSN_NP_63
AGCAAAAGCAGGGTAGATAATCACTCACAGAGTGACATCGAGAAAAATACCCTTGTTTCTACT
>WSN_NP_88
AGCAAAAGCAGGGTAGATAATCACTCACAGAGTGACATCGCAATGCAGAGGAGTACGACAATTAAAGAAAAATACCCTTGTTTC
TACT
>WSN_NP_60
AGCAAAAGCAGGGTAGATAATCACTCACAGAGTGACATCAAAAATACCCTTGTTTCTACT
>WSN_NP_64
AGCAAAAGCAGGGTAGATAATCACTCACAGAGTGACATCAAAGAAAAATACCCTTGTTTCTACT
>WSN_NP_84
AGCAAAAGCAGGGTAGATAATCACTCACAGAGTGACATATGCAGAGGAGTACGACAATTAAAGAAAAATACCCTTGTTTCTACT
>WSN_NP_59
AGCAAAAGCAGGGTAGATAATCACTCACAGAGTGACAGAAAAATACCCTTGTTTCTACT
>WSN_NP_71
AGCAAAAGCAGGGTAGATAATCACTCACAGAGTGACAACGACAATTAAAGAAAAATACCCTTGTTTCTACT
>WSN_NP_64
AGCAAAAGCAGGGTAGATAATCACTCACAGAGTGAAATTAAAGAAAAATACCCTTGTTTCTACT
>WSN_NP_71
AGCAAAAGCAGGGTAGATAATCACTCACAGAGTGAGTACGACAATTAAAGAAAAATACCCTTGTTTCTACT
>WSN_NP_63
AGCAAAAGCAGGGTAGATAATCACTCACAGAGTCAATTAAAGAAAAATACCCTTGTTTCTACT
>WSN_NP_50
AGCAAAAGCAGGGTAGATAATCACTAAAGAAAAATACCCTTGTTTCTACT
>WSN_NP_62
AGCAAAAGCAGGGTAGATAATCACGAGTACGACAATTAAAGAAAAATACCCTTGTTTCTACT
>WSN_NP_45
AGCAAAAGCAGGGTAGATAATCAGAAAAATACCCTTGTTTCTACT
>WSN_NP_50
AGCAAAAGCAGGGTAGATAATCATTAAAGAAAAATACCCTTGTTTCTACT
>WSN_NP_42
AGCAAAAGCAGGGTAGATAAGAAAAATACCCTTGTTTCTACT
>WSN_NP_46
AGCAAAAGCAGGGTAGATATTAAAGAAAAATACCCTTGTTTCTACT
>WSN_NP_46
AGCAAAAGCAGGGTAGAAATTAAAGAAAAATACCCTTGTTTCTACT
>WSN_NA_86
AGCAAAAGCAGGAGTTTAAATGAATCCAAACCAGAAAATAATAACCATTGGaTCgATCTGTATGCAAAAAACTCCTTGTTTCTACT
>WSN_NA_64
AGCAAAAGCAGGAGTTTAAATGAATCCAAACCAGAAAATAATAACAAAACTCCTTGTTTCTACT
>WSN_NA_66
AGCAAAAGCAGGAGTTTAAATGAATCCAAACCAGATAGTTTGTTCAAAAAACTCCTTGTTTCTACT
>WSN_NA_67
AGCAAAAGCAGGAGTTTAAATGAATCCAAACCAAAGTAGTTTGTTCAAAAAACTCCTTGTTTCTACT
>WSN_NA_68
AGCAAAAGCAGGAGTTTAAATGAATCCAAACCACAAGTAGTTTGTTCAAAAAACTCCTTGTTTCTACT
>WSN_NA_69
AGCAAAAGCAGGAGTTTAAATGAATCCAAACCAACAAGTAGTTTGTTCAAAAAACTCCTTGTTTCTACT
>WSN_NA_72
AGCAAAAGCAGGAGTTTAAATGAATCCAAACCATTGACAAGTAGTTTGTTCAAAAAACTCCTTGTTTCTACT
```

>WSN_NA_63
AGCAAAAGCAGGAGTTTAAATGAATCCAAACCTAGTTTGTTCAAAAAACTCCTTGTTTCTACT
>WSN_NA_69
AGCAAAAGCAGGAGTTTAAATGAATCCAAACCGACAAGTAGTTTGTTCAAAAAACTCCTTGTTTCTACT
>WSN_NA_65
AGCAAAAGCAGGAGTTTAAATGAATCCAAACAAGTAGTTTGTTCAAAAAACTCCTTGTTTCTACT
>WSN_NA_66
AGCAAAAGCAGGAGTTTAAATGAATCCAAAACAAGTAGTTTGTTCAAAAAACTCCTTGTTTCTACT
>WSN_NA_67
AGCAAAAGCAGGAGTTTAAATGAATCCAAAGACAAGTAGTTTGTTCAAAAAACTCCTTGTTTCTACT
>WSN_NA_68
AGCAAAAGCAGGAGTTTAAATGAATCCAAATGACAAGTAGTTTGTTCAAAAAACTCCTTGTTTCTACT
>WSN_NA_65
AGCAAAAGCAGGAGTTTAAATGAATCCAGACAAGTAGTTTGTTCAAAAAACTCCTTGTTTCTACT
>WSN_NA_68
AGCAAAAGCAGGAGTTTAAATGAATCCCATTGACAAGTAGTTTGTTCAAAAAACTCCTTGTTTCTACT
>WSN_NA_55
AGCAAAAGCAGGAGTTTAAATGAGTAGTTTGTTCAAAAAACTCCTTGTTTCTACT
>WSN_NA_63
AGCAAAAGCAGGAGTTTAAATGCATTGACAAGTAGTTTGTTCAAAAAACTCCTTGTTTCTACT
>WSN_NA_49
AGCAAAAGCAGGAGTTTAAATTTTGTTCAAAAAACTCCTTGTTTCTACT
>WSN_NA_56
AGCAAAAGCAGGAGTTTAAATCAAGTAGTTTGTTCAAAAAACTCCTTGTTTCTACT
>WSN_NA_43
AGCAAAAGCAGGAGTTTAATTCAAAAAACTCCTTGTTTCTACT
>WSN_NA_62
AGCAAAAGCAGGAGTTTACACCATTGACAAGTAGTTTGTTCAAAAAACTCCTTGTTTCTACT
>WSN_M_78
AGCGAAAGCAGGTAGATATTGAAAGATGAGTCTTCTAACCGAGGTCGAAACGGAGTAAAAAACTACCTTGTTTCTACT
>WSN_M_61
AGCGAAAGCAGGTAGATATTGAAAGATTAGAGCTGGAGTAAAAAACTACCTTGTTTCTACT
>WSN_M_50
AGCGAAAGCAGGTAGATATTGAAAGATTAAAAAACTACCTTGTTTCTACT
>WSN_M_43
AGCGAAAGCAGGTAGATATTTAAAAAACTACCTTGTTTCTACT
>WSN_M_46
AGCGAAAGCAGGTAGATATGGAGTAAAAAACTACCTTGTTTCTACT
>WSN_HA_77
AGCAAAAGCAGGGGAAAATAAAAACAACCAAAATGTAGGATTTCAGAAATATAAGGAAAAACACCCTTGTTTCTACT
>WSN_HA_53
AGCAAAAGCAGGGGAAAATAAAAACAACCAAAATAAACACCCTTGTTTCTACT
>WSN_HA_61
AGCAAAAGCAGGGGAAAATAAAAACAACCAAAATATAAGGAAAAACACCCTTGTTTCTACT
>WSN_HA_65
AGCAAAAGCAGGGGAAAATAAAAACAACCAAAATAAATATAAGGAAAAACACCCTTGTTTCTACT
>WSN_HA_60
AGCAAAAGCAGGGGAAAATAAAAACAACCAAAAATAAGGAAAAACACCCTTGTTTCTACT
>WSN_HA_87
AGCAAAAGCAGGGGAAAATAAAAACAACCAATATGCATCTGAGATTAGGATTTCAGAAATATAAGGAAAAACACCCTTGTTTCTA
CT
>WSN_HA_65
AGCAAAAGCAGGGGAAAATAAAAACAACCATCAGAAATATAAGGAAAAACACCCTTGTTTCTACT
>WSN_HA_66
AGCAAAAGCAGGGGAAAATAAAAACAACCATTCAGAAATATAAGGAAAAACACCCTTGTTTCTACT
>WSN_HA_51
AGCAAAAGCAGGGGAAAATAAAAACAACCGAAAAACACCCTTGTTTCTACT
>WSN_HA_50
AGCAAAAGCAGGGGAAAATAAAAACAACGAAAAACACCCTTGTTTCTACT
>WSN_HA_46
AGCAAAAGCAGGGGAAAATAAAAACAAAAACACCCTTGTTTCTACT
>WSN_HA_65
AGCAAAAGCAGGGGAAAATAAAAACAGATTTCAGAAATATAAGGAAAAACACCCTTGTTTCTACT
>WSN_HA_44
AGCAAAAGCAGGGGAAAATAAAAAAAAACACCCTTGTTTCTACT
>WSN_HA_56
AGCAAAAGCAGGGGAAAATAAAAAGAAATATAAGGAAAAACACCCTTGTTTCTACT
>WSN_HA_60
AGCAAAAGCAGGGGAAAATAAAAATTCAGAAATATAAGGAAAAACACCCTTGTTTCTACT
>WSN_HA_63

AGCAAAAGCAGGGGAAAATAAAAAGATTTCAGAAATATAAGGAAAAACACCCTTGTTTCTACT
>WSN_HA_44
AGCAAAAGCAGGGGAAAATAAGGAAAAACACCCTTGTTTCTACT
>WSN_HA_60
AGCAAAAGCAGGGGAAAATAGGATTTCAGAAATATAAGGAAAAACACCCTTGTTTCTACT
>WSN_HA_53
AGCAAAAGCAGGGGAAAATCAGAAATATAAGGAAAAACACCCTTGTTTCTACT
>WSN_HA_58
AGCAAAAGCAGGGGAAAATGATTTCAGAAATATAAGGAAAAACACCCTTGTTTCTACT
>WSN_HA_41
AGCAAAAGCAGGGGAAAAGGAAAAACACCCTTGTTTCTACT
>WSN_HA_43
AGCAAAAGCAGGGGAAAAAAGGAAAAACACCCTTGTTTCTACT
>WSN_HA_50
AGCAAAAGCAGGGGAAAAGAAATATAAGGAAAAACACCCTTGTTTCTACT
>WSN_HA_64
AGCAAAAGCAGGGGAAAAAGATTAGGATTTCAGAAATATAAGGAAAAACACCCTTGTTTCTACT

Supplemental Fasta file 2. WSN mvRNA sequences.

>WSN_NP_61
AGCAAAAGCAGGGTAGATAATCACTCACAGAGTGACATAGAAAAATACCCTTGTTTCTACT
>WSN_PB1_57
AGCGAAAGCAGGCAAACCATTTGAATGGATTTCATGAAAAAATGCCTTGTTTCTACT
>WSN_PB1_57
AGCGAAAGCAGGCAAACCATTTGAATGTCCTTCATGAAAAAATGCCTTGTTTCTACT
>WSN_PB1_62
AGCGAAAGCAGGCAAACCATTTGAATGGATGTCAATCCGACTTAAAATGCCTTGTTTCTACT
>WSN_PB1_66
AGCGAAAGCAGGCAAACCATTTGAATGTTTAGCTTGTCCTTCATGAAAAAATGCCTTGTTTCTACT
>WSN_NP_58
AGCAAAAGCAGGGTAGATAATCACTCACAGAGTGACATCAAATACCCTTGTTTCTACT
>WSN_HA_60
AGCAAAAGCAGGGGAAATTAGGATTTCAGAAATATAAGGAAAAACACCCTTGTTTCTACT
>WSN_HA_44
AGCAAAAGCAGGGGAATATAAGGAAAAACACCCTTGTTTCTACT
>WSN_PB1_69
AGCGAAAGCAGGCAAACCATTTGAATGGATGTTAGCTTGTCCTTCATGAAAAAATGCCTTGTTTCTACT
>WSN_PB1_61
AGCGAAAGCAGGCAAACCATTTGAATGGTTGTCCTTCATGAAAAAATGCCTTGTTTCTACT
>WSN_NP_63
AGCAAAAGCAGGGTAGATAATCACTCACAGAGTGACTTAAAGAAAAATACCCTTGTTTCTACT
>WSN_NA_70
AGCAAAAGCAGGAGTTTAAATGAATCCAAACCTGACAAGTAGTTTGTTCAAAAAACTCCTTGTTTCTACT
>WSN_NA_62
AGCAAAAGCAGGAGTTTACACCATTGACAAGTAGTTTGTTCAAAAAACTCCTTGTTTCTACT
>WSN_PB1_65
AGCGAAAGCAGGCAAACCATTTGAATGGATGTCAATCCGACTTGAAAAAATGCCTTGTTTCTACT
>WSN_PB1_69
AGCGAAAGCAGGCAAACCATTTGAATGGATGTCAATCCGTCCTTCATGAAAAAATGCCTTGTTTCTACT
>WSN_PB1_48
AGCGAAAGCAGGCAAACCATTTGAATGAAAAAATGCCTTGTTTCTACT
>WSN_PB1_62
AGCGAAAGCAGGCAAACCATTTGAATAGCTTGTCCTTCATGAAAAAATGCCTTGTTTCTACT
>WSN_NP_71
AGCAAAAGCAGGGTAGATAATCACTCACAGAGTGACATCGAAATCATGGCAAAAATACCCTTGTTTCTACT
>WSN_NP_76
AGCAAAAGCAGGGTAGATAATCACTCACAGAGTGACATCGAAATCATGGCTAAAGAAAAATACCCTTGTTTCTACT
>WSN_NP_64
AGCAAAAGCAGGGTAGATAATCACTCACAGAGTGACATCGAAATAAAATACCCTTGTTTCTACT
>WSN_NP_55
AGCAAAAGCAGGGTAGATAATCACTCACAGAGAGAAAAATACCCTTGTTTCTACT
>WSN_NP_44
AGCAAAAGCAGGGTAGATTAAAGAAAAATACCCTTGTTTCTACT
>WSN_HA_58
AGCAAAAGCAGGGGAATAGGATTTCAGAAATATAAGGAAAAACACCCTTGTTTCTACT
>WSN_HA_64
AGCAAAAGCAGGGGAATGAGATTAGGATTTCAGAAATATAAGGAAAAACACCCTTGTTTCTACT
>WSN_PB2_80
AGCGAAAGCAGGTCAATTATATTCAATATGGAAGGCCATCAATTAGTGTCGAATAGTTTAAAAACGACCTTGTTTCTACT
>WSN_PB1_77
AGCGAAAGCAGGCAAACCATTTGAATGGATGTCAATCCGACTTTACTTTTCTCATGAAAAAATGCCTTGTTTCTACT
>WSN_PB1_65
AGCGAAAGCAGGCAAACCATTTGAATGGATGTCAATCCGACTTTACAAAATGCCTTGTTTCTACT
>WSN_PB1_64
AGCGAAAGCAGGCAAACCATTTGAATGGATGTCAATCCGACTTAAAAAATGCCTTGTTTCTACT
>WSN_PB1_68
AGCGAAAGCAGGCAAACCATTTGAATGGATGTCAATCCGACTTCATGAAAAAATGCCTTGTTTCTACT
>WSN_PB1_64
AGCGAAAGCAGGCAAACCATTTGAATGGATGTCAATCCGACTGAAAAAATGCCTTGTTTCTACT
>WSN_PB1_58
AGCGAAAGCAGGCAAACCATTTGAATGGATGTCAATCC AAAAATGCCTTGTTTCTACT
>WSN_PB1_64
AGCGAAAGCAGGCAAACCATTTGAATGGATGTCAATCCTCATGAAAAAATGCCTTGTTTCTACT
>WSN_PB1_68
AGCGAAAGCAGGCAAACCATTTGAATGGATGTCAATCCTCCTTCATGAAAAAATGCCTTGTTTCTACT
>WSN_PB1_60
AGCGAAAGCAGGCAAACCATTTGAATGGATGTCAATCTGAAAAAATGCCTTGTTTCTACT
>WSN_PB1_54

AGCGAAAGCAGGCAAACCATTTGAATGGATGTGAAAAAATGCCTTGTTTCTACT
>WSN_PB1_50
AGCGAAAGCAGGCAAACCATTTGAATGGGAAAAAATGCCTTGTTTCTACT
>WSN_PB1_66
AGCGAAAGCAGGCAAACCATTTGAATGGTTAGCTTGTCCTTCATGAAAAAATGCCTTGTTTCTACT
>WSN_PB1_49
AGCGAAAGCAGGCAAACCATTTGAATTGAAAAAATGCCTTGTTTCTACT
>WSN_PB1_48
AGCGAAAGCAGGCAAACCATTTGCATGAAAAAATGCCTTGTTTCTACT
>WSN_PB1_43
AGCGAAAGCAGGCAAACCATTTAAAAAATGCCTTGTTTCTACT
>WSN_PB1_64
AGCGAAAGCAGGCAAACCATGTGAATTTAGCTTGTCCTTCATGAAAAAATGCCTTGTTTCTACT
>WSN_PB1_61
AGCGAAAGCAGGCAAACCTGAATTTAGCTTGTCCTTCATGAAAAAATGCCTTGTTTCTACT
>WSN_PB1_40
AGCGAAAGCAGGCAAACTGAAAAAATGCCTTGTTTCTACT
>WSN_PB1_56
AGCGAAAGCAGGCAAACTTTAGCTTGTCCTTCATGAAAAAATGCCTTGTTTCTACT
>WSN_PA_60
AGCGAAAGCAGGTACTGATTCAAAATGGCATACTGTCCAAAAAAGTACCTTGTTTCTACT
>WSN_PA_66
AGCGAAAGCAGGTACTGATTCAAAATGTGCTATCCATACTGTCCAAAAAAGTACCTTGTTTCTACT
>WSN_NS_80
AGCAAAAGCAGGGTGACAAAGACATAATGGATCCAAACACTGTGTCAAGCTTTCAGATAAAAAACACCCTTGTTTCTACT
>WSN_NS_56
AGCAAAAGCAGGGTGACAAAGACATAATGGATCTAAAAAACACCCTTGTTTCTACT
>WSN_NS_55
AGCAAAAGCAGGGTGACAAAGACATAATGTAATAAAAAACACCCTTGTTTCTACT
>WSN_NP_66
AGCAAAAGCAGGGTAGATAATCACTCACAGAGTGACATCGTAAAGAAAAATACCCTTGTTTCTACT
>WSN_NP_60
AGCAAAAGCAGGGTAGATAATCACTCACAGAGTGACATCAAAAATACCCTTGTTTCTACT
>WSN_NP_65
AGCAAAAGCAGGGTAGATAATCACTCACAGAGTGACATCTAAAGAAAAATACCCTTGTTTCTACT
>WSN_NP_66
AGCAAAAGCAGGGTAGATAATCACTCACAGAGTGACATCTTAAAGAAAAATACCCTTGTTTCTACT
>WSN_NP_74
AGCAAAAGCAGGGTAGATAATCACTCACAGAGTGACATCTACGACAATTAAAGAAAAATACCCTTGTTTCTACT
>WSN_NP_64
AGCAAAAGCAGGGTAGATAATCACTCACAGAGTGACATTAAAGAAAAATACCCTTGTTTCTACT
>WSN_NP_59
AGCAAAAGCAGGGTAGATAATCACTCACAGAGTGACAGAAAAATACCCTTGTTTCTACT
>WSN_NP_72
AGCAAAAGCAGGGTAGATAATCACTCACAGAGTGACGTACGACAATTAAAGAAAAATACCCTTGTTTCTACT
>WSN_NP_70
AGCAAAAGCAGGGTAGATAATCACTCACAGAGTGATACGACAATTAAAGAAAAATACCCTTGTTTCTACT
>WSN_NP_57
AGCAAAAGCAGGGTAGATAATCACTCACAGAGTAAGAAAAATACCCTTGTTTCTACT
>WSN_NP_44
AGCAAAAGCAGGGTAGATAATCACTAAATACCCTTGTTTCTACT
>WSN_NP_49
AGCAAAAGCAGGGTAGATAATCACTAAGAAAAATACCCTTGTTTCTACT
>WSN_NP_43
AGCAAAAGCAGGGTAGTTAAAGAAAAATACCCTTGTTTCTACT
>WSN_NA_75
AGCAAAAGCAGGAGTTTAAATGAATCCAAACCAGAAAATAAAAGTAGTTTGTTCAAAAAACTCCTTGTTTCTACT
>WSN_NA_73
AGCAAAAGCAGGAGTTTAAATGAATCCAAACCAGAAAACAAGTAGTTTGTTCAAAAAACTCCTTGTTTCTACT
>WSN_NA_79
AGCAAAAGCAGGAGTTTAAATGAATCCAAACCAGAAAACATTGACAAGTAGTTTGTTCAAAAAACTCCTTGTTTCTACT
>WSN_NA_69
AGCAAAAGCAGGAGTTTAAATGAATCCAAACCAGAAAGTAGTTTGTTCAAAAAACTCCTTGTTTCTACT
>WSN_NA_61
AGCAAAAGCAGGAGTTTAAATGAATCCAAACCAGATGTTCAAAAAACTCCTTGTTTCTACT
>WSN_NA_60
AGCAAAAGCAGGAGTTTAAATGAATCCAAACCAGTGTTCAAAAAACTCCTTGTTTCTACT
>WSN_NA_69
AGCAAAAGCAGGAGTTTAAATGAATCCAAACCAACAAGTAGTTTGTTCAAAAAACTCCTTGTTTCTACT
>WSN_NA_66

AGCAAAAGCAGGAGTTTAAATGAATCCAAAACAAGTAGTTTGTTCAAAAAACTCCTTGTTTCTACT
>WSN_NA_60
AGCAAAAGCAGGAGTTTAAATGAATCCAATAGTTTGTTCAAAAAACTCCTTGTTTCTACT
>WSN_NA_63
AGCAAAAGCAGGAGTTTAACACCATTGACAAGTAGTTTGTTCAAAAAACTCCTTGTTTCTACT
>WSN_NA_62
AGCAAAAGCAGGAGTTTTCACCATTGACAAGTAGTTTGTTCAAAAAACTCCTTGTTTCTACT
>WSN_NA_63
AGCAAAAGCAGGAGTCGTTCACCATTGACAAGTAGTTTGTTCAAAAAACTCCTTGTTTCTACT
>WSN_M_71
AGCGAAAGCAGGTAGATATTGAAAGATGAGTCTTCCATAGAGCTGGAGTAAAAAACTACCTTGTTTCTACT
>WSN_M_52
AGCGAAAGCAGGTAGATATTGAAAGATGAGAAAAAACTACCTTGTTTCTACT
>WSN_M_48
AGCGAAAGCAGGTAGATATTGAAAGATAAAAACTACCTTGTTTCTACT
>WSN_M_47
AGCGAAAGCAGGTAGATATTGAAATAAAAAACTACCTTGTTTCTACT
>WSN_HA_89
AGCAAAAGCAGGGGAAAATAAAAACAACCAAAATGAAGGCAGATTAGGATTTCAGAAATATAAGGAAAAACACCCTTGTTTCTAC
T
>WSN_HA_90
AGCAAAAGCAGGGGAAAATAAAAACAACCAAAATGAAGGCCTGAGATTAGGATTTCAGAAATATAAGGAAAAACACCCTTGTTTC
TACT
>WSN_HA_50
AGCAAAAGCAGGGGAAAATAAAAACAACGAAAAACACCCTTGTTTCTACT
>WSN_HA_63
AGCAAAAGCAGGGGAAAATAAAAACATTTCAGAAATATAAGGAAAAACACCCTTGTTTCTACT
>WSN_HA_44
AGCAAAAGCAGGGGAAAATAAAGAAAAACACCCTTGTTTCTACT
>WSN_HA_41
AGCAAAAGCAGGGGAAAATGAAAAACACCCTTGTTTCTACT
>WSN_HA_65
AGCAAAAGCAGGGGAAATGAGATTAGGATTTCAGAAATATAAGGAAAAACACCCTTGTTTCTACT
>WSN_HA_66
AGCAAAAGCAGGGGAAACTGAGATTAGGATTTCAGAAATATAAGGAAAAACACCCTTGTTTCTACT
>WSN_HA_61
AGCAAAAGCAGGGGAAGATTAGGATTTCAGAAATATAAGGAAAAACACCCTTGTTTCTACT

Supplemental Fasta file 3. BM18 mvRNA sequences.

>BM18_PB2,1,2325,35:(18+17)
AGCRAAAGCAGGTCAATTACGACCTTGTTTCTACT
>BM18_M,1,1009,36:(17+19)
AGCRAAAGCAGGTAGATAAACTACCTTGTTTCTACT
>BM18_M,1,1010,35:(17+18)
AGCRAAAGCAGGTAGATAACTACCTTGTTTCTACT
>BM18_PB2,1,2324,36:(18+18)
AGCRAAAGCAGGTCAATTAACGACCTTGTTTCTACT
>BM18_NS,1,872,36:(17+19)
AGCRAAAGCAGGGTGACAAACACCCTTGTTTCTACT
>BM18_NP,1,1547,37:(18+19)
AGCRAAAGCAGGGTAGATAAATACCCTTGTTTCTACT
>BM18_HA,1,1762,36:(19+17)
AGCRAAAGCAGGGGAAAATACACCCTTGTTTCTACT
>BM18_NP,1,1541,41:(16+25)
AGCRAAAGCAGGGTAGAAAGAAAAATACCCTTGTTTCTACT
>BM18_NP,1,1547,35:(16+19)
AGCRAAAGCAGGGTAGAAATACCCTTGTTTCTACT
>BM18_PB2,1,2323,37:(18+19)
AGCRAAAGCAGGTCAATTAAACGACCTTGTTTCTACT
>BM18_PB2,1,2319,39:(16+23)
AGCRAAAGCAGGTCAATTAAAAACGACCTTGTTTCTACT
>BM18_HA,1,1761,37:(19+18)
AGCRAAAGCAGGGGAAAATAACACCCTTGTTTCTACT
>BM18_NS,1,869,39:(17+22)
AGCRAAAGCAGGGTGACAAAAAACACCCTTGTTTCTACT
>BM18_NP,1,1545,39:(18+21)
AGCRAAAGCAGGGTAGATAAAAATACCCTTGTTTCTACT
>BM18_NP,1,1548,34:(16+18)
AGCRAAAGCAGGGTAGAATACCCTTGTTTCTACT
>BM18_M,1,1008,37:(17+20)
AGCRAAAGCAGGTAGATAAAACTACCTTGTTTCTACT
>BM18_NS,1,871,37:(17+20)
AGCRAAAGCAGGGTGACAAAACACCCTTGTTTCTACT
>BM18_NS,1,873,35:(17+18)
AGCRAAAGCAGGGTGACAACACCCTTGTTTCTACT
>BM18_HA,1,1760,38:(19+19)
AGCRAAAGCAGGGGAAAATAAACACCCTTGTTTCTACT
>BM18_HA,1,1752,44:(17+27)
AGCRAAAGCAGGGGAAAATAAGGAAAAACACCCTTGTTTCTACT
>BM18_NP,1,1546,36:(16+20)
AGCRAAAGCAGGGTAGAAAATACCCTTGTTTCTACT
>BM18_M,1,1005,39:(16+23)
AGCRAAAGCAGGTAGATAAAAAACTACCTTGTTTCTACT
>BM18_NP,1,1546,38:(18+20)
AGCRAAAGCAGGGTAGATAAAATACCCTTGTTTCTACT
>BM18_PB1,1,2324,36:(18+18)
AGCRAAAGCAGGCAAACCAAATGCCTTGTTTCTACT
>BM18_HA,1,1757,41:(19+22)
AGCRAAAGCAGGGGAAAATGAAAAACACCCTTGTTTCTACT
>BM18_NP,1,1540,43:(17+26)
AGCRAAAGCAGGGTAGATAAAGAAAAATACCCTTGTTTCTACT
>BM18_NP,1,1544,38:(16+22)
AGCRAAAGCAGGGTAGGAAAAATACCCTTGTTTCTACT
>BM18_PB1,1,2323,37:(18+19)
AGCRAAAGCAGGCAAACCAAAATGCCTTGTTTCTACT
>BM18_HA,1,1758,37:(16+21)
AGCRAAAGCAGGGGAAAAAAACACCCTTGTTTCTACT
>BM18_NP,1,1549,35:(18+17)
AGCRAAAGCAGGGTAGATATACCCTTGTTTCTACT
>BM18_PB2,1,2322,38:(18+20)
AGCRAAAGCAGGTCAATTAAAACGACCTTGTTTCTACT
>BM18_M,1,1011,34:(17+17)
AGCRAAAGCAGGTAGATACTACCTTGTTTCTACT
>BM18_HA,1,1759,39:(19+20)
AGCRAAAGCAGGGGAAAATAAAACACCCTTGTTTCTACT
>BM18_PB2,1,2315,44:(17+27)
AGCRAAAGCAGGTCAATTAGTTTAAAAACGACCTTGTTTCTACT
>BM18_NP,1,1543,39:(16+23)

AGCRAAAGCAGGGTAGAGAAAAATACCCTTGTTTCTACT
>BM18_HA,1,1758,38:(17+21)
AGCRAAAGCAGGGGAAAAAAAACACCCTTGTTTCTACT
>BM18_HA,1,1754,42:(17+25)
AGCRAAAGCAGGGGAAAAAGGAAAAACACCCTTGTTTCTACT
>BM18_PB1,1,2325,35:(18+17)
AGCRAAAGCAGGCAAACCAATGCCTTGTTTCTACT
>BM18_NS,1,867,47:(23+24)
AGCRAAAGCAGGGTGACAAAGACATAAAAAACACCCTTGTTTCTACT
>BM18_NS,1,870,38:(17+21)
AGCRAAAGCAGGGTGACAAAAACACCCTTGTTTCTACT
>BM18_NS,1,874,34:(17+17)
AGCRAAAGCAGGGTGACACACCCTTGTTTCTACT
>BM18_PB1,1,2321,39:(18+21)
AGCRAAAGCAGGCAAACCAAAAAATGCCTTGTTTCTACT
>BM18_M,1,1007,38:(17+21)
AGCRAAAGCAGGTAGATAAAAACTACCTTGTTTCTACT
>BM18_HA,1,1756,42:(19+23)
AGCRAAAGCAGGGGAAAATGGAAAAACACCCTTGTTTCTACT
>BM18_NA,1,1439,36:(17+19)
AGCRAAAGCAGGAGTTTAAAACTCCTTGTTTCTACT
>BM18_PB1,1,2322,38:(18+20)
AGCRAAAGCAGGCAAACCAAAAATGCCTTGTTTCTACT
>BM18_M,1,1004,41:(17+24)
AGCRAAAGCAGGTAGATGTAAAAAACTACCTTGTTTCTACT
>BM18_NP,1,1544,40:(18+22)
AGCRAAAGCAGGGTAGATGAAAAATACCCTTGTTTCTACT
>BM18_NP,1,1542,40:(16+24)
AGCRAAAGCAGGGTAGAAGAAAAATACCCTTGTTTCTACT
>BM18_HA,1,1754,41:(16+25)
AGCRAAAGCAGGGGAAAAGGAAAAACACCCTTGTTTCTACT
>BM18_PB2,1,2313,47:(18+29)
AGCRAAAGCAGGTCAATTAATAGTTTAAAAACGACCTTGTTTCTACT
>BM18_M,1,1005,42:(19+23)
AGCRAAAGCAGGTAGATGTTAAAAAACTACCTTGTTTCTACT
>BM18_NP,1,1543,41:(18+23)
AGCRAAAGCAGGGTAGATAGAAAAATACCCTTGTTTCTACT
>BM18_PB2,1,2313,53:(24+29)
AGCRAAAGCAGGTCAATTATATTCAATAGTTTAAAAACGACCTTGTTTCTACT
>BM18_PB2,1,2314,46:(18+28)
AGCRAAAGCAGGTCAATTATAGTTTAAAAACGACCTTGTTTCTACT
>BM18_PB1,1,2322,43:(23+20)
AGCRAAAGCAGGCAAACCATTTGAAAAATGCCTTGTTTCTACT
>BM18_PB1,1,2319,44:(21+23)
AGCRAAAGCAGGCAAACCATTTGAAAAAATGCCTTGTTTCTACT
>BM18_M,1,1006,43:(21+22)
AGCRAAAGCAGGTAGATGTTGAAAAAACTACCTTGTTTCTACT
>BM18_PA,1,2212,48:(26+22)
AGCRAAAGCAGGTACTGATTCAAAATAAAAAAGTACCTTGTTTCTACT
>BM18_PA,1,2216,39:(21+18)
AGCRAAAGCAGGTACTGATTCAAGTACCTTGTTTCTACT
>BM18_PA,1,2216,34:(16+18)
AGCRAAAGCAGGTACTAAGTACCTTGTTTCTACT
>BM18_PA,1,2215,35:(16+19)
AGCRAAAGCAGGTACTAAAGTACCTTGTTTCTACT
>BM18_PB2,1,2308,60:(26+34)
AGCRAAAGCAGGTCAATTATATTCAATGTCGAATAGTTTAAAAACGACCTTGTTTCTACT
>BM18_HA,1,1746,52:(19+33)
AGCRAAAGCAGGGGAAAATAGAAATATAAGGAAAAACACCCTTGTTTCTACT
>BM18_HA,1,1741,57:(19+38)
AGCRAAAGCAGGGGAAAATATTTCAGAAATATAAGGAAAAACACCCTTGTTTCTACT
>BM18_HA,1,1758,39:(18+21)
AGCRAAAGCAGGGGAAAAAAAAACACCCTTGTTTCTACT
>BM18_HA,1,1737,60:(18+42)
AGCRAAAGCAGGGGAAAATAGGATTTCAGAAATATAAGGAAAAACACCCTTGTTTCTACT
>BM18_NS,1,869,43:(21+22)
AGCRAAAGCAGGGTGACAAAGAAAAAACACCCTTGTTTCTACT
>BM18_M,1,1000,46:(18+28)
AGCRAAAGCAGGTAGATGTGGAGTAAAAAACTACCTTGTTTCTACT
>BM18_NA,1,1409,66:(17+49)

AGCRAAAGCAGGAGTTTCCATTCACCATTGACAAGTAGTTTGTTCAAAAAACTCCTTGTTTCTACT
>BM18_NA,1,1423,51:(16+35)
AGCRAAAGCAGGAGTTCAAGTAGTTTGTTCAAAAAACTCCTTGTTTCTACT
>BM18_NP,1,1545,38:(17+21)
AGCRAAAGCAGGGTAGAAAAAATACCCTTGTTTCTACT
>BM18_NP,1,1541,42:(17+25)
AGCRAAAGCAGGGTAGAAAAGAAAAATACCCTTGTTTCTACT
>BM18_PA,1,2207,52:(25+27)
AGCRAAAGCAGGTACTGATTCAAAATGTCCAAAAAAGTACCTTGTTTCTACT
>BM18_PA,1,2211,43:(20+23)
AGCRAAAGCAGGTACTGATTCAAAAAAGTACCTTGTTTCTACT
>BM18_PB1,1,2320,45:(23+22)
AGCRAAAGCAGGCAAACCATTTGGAAAAAATGCCTTGTTTCTACT
>BM18_PB1,1,2302,58:(18+40)
AGCRAAAGCAGGCAAACCATTTAGCTTGTCCTTCATGAAAAAATGCCTTGTTTCTACT
>BM18_HA,1,1755,43:(19+24)
AGCRAAAGCAGGGGAAAATAGGAAAAACACCCTTGTTTCTACT
>BM18_HA,1,1757,38:(16+22)
AGCRAAAGCAGGGGAAGAAAAACACCCTTGTTTCTACT
>BM18_NS,1,864,50:(23+27)
AGCRAAAGCAGGGTGACAAAGACATAATAAAAAACACCCTTGTTTCTACT
>BM18_NS,1,869,42:(20+22)
AGCRAAAGCAGGGTGACAAAAAAAAACACCCTTGTTTCTACT
>BM18_M,1,988,73:(33+40)
AGCRAAAGCAGGTAGATGTTGAAAGATGAGTCTTCAACATAGAGCTGGAGTAAAAAACTACCTTGTTTCTACT
>BM18_NA,1,1428,62:(32+30)
AGCRAAAGCAGGAGTTTAAATGAATCCAAATCAGTTTGTTCAAAAAACTCCTTGTTTCTACT
>BM18_NA,1,1437,38:(17+21)
AGCRAAAGCAGGAGTTTAAAAAACTCCTTGTTTCTACT
>BM18_NP,1,1547,58:(39+19)
AGCRAAAGCAGGGTAGATAATCACTCACTGAGTGACATCAAATACCCTTGTTTCTACT
>BM18_NP,1,1546,56:(36+20)
AGCRAAAGCAGGGTAGATAATCACTCACTGAGTGACAAAATACCCTTGTTTCTACT
>BM18_NP,1,1535,59:(28+31)
AGCRAAAGCAGGGTAGATAATCACTCACACAATTAAAGAAAAATACCCTTGTTTCTACT
>BM18_NP,1,1540,42:(16+26)
AGCRAAAGCAGGGTAGTAAAGAAAAATACCCTTGTTTCTACT
>BM18_PA,1,2213,42:(21+21)
AGCRAAAGCAGGTACTGATTCAAAAAGTACCTTGTTTCTACT
>BM18_PA,1,2216,35:(17+18)
AGCRAAAGCAGGTACTGAAGTACCTTGTTTCTACT
>BM18_PA,1,2217,33:(16+17)
AGCRAAAGCAGGTACTAGTACCTTGTTTCTACT
>BM18_PB2,1,2312,48:(18+30)
AGCRAAAGCAGGTCAATTGAATAGTTTAAAAACGACCTTGTTTCTACT
>BM18_PB2,1,2308,51:(17+34)
AGCRAAAGCAGGTCAATTGTCGAATAGTTTAAAAACGACCTTGTTTCTACT
>BM18_PB1,1,2296,71:(25+46)
AGCRAAAGCAGGCAAACCATTTGAATAGTGAATTTAGCTTGTCCTTCATGAAAAAATGCCTTGTTTCTACT
>BM18_PB1,1,2324,40:(22+18)
AGCRAAAGCAGGCAAACCATTTAAATGCCTTGTTTCTACT
>BM18_PB1,1,2321,43:(22+21)
AGCRAAAGCAGGCAAACCATTTAAAAAATGCCTTGTTTCTACT
>BM18_HA,1,1758,46:(25+21)
AGCRAAAGCAGGGGAAAATAAAAACAAAAACACCCTTGTTTCTACT
>BM18_HA,1,1746,55:(22+33)
AGCRAAAGCAGGGGAAAATAAAAGAAATATAAGGAAAAACACCCTTGTTTCTACT
>BM18_HA,1,1731,66:(18+48)
AGCRAAAGCAGGGGAAAATGAGATTAGGATTTCAGAAATATAAGGAAAAACACCCTTGTTTCTACT
>BM18_HA,1,1757,39:(17+22)
AGCRAAAGCAGGGGAAAGAAAAACACCCTTGTTTCTACT
>BM18_NS,1,873,43:(25+18)
AGCRAAAGCAGGGTGACAAAGACATAACACCCTTGTTTCTACT
>BM18_NS,1,870,46:(25+21)
AGCRAAAGCAGGGTGACAAAGACATAAAAACACCCTTGTTTCTACT
>BM18_NS,1,858,57:(24+33)
AGCRAAAGCAGGGTGACAAAGACATATTTAATAATAAAAAACACCCTTGTTTCTACT
>BM18_NS,1,872,42:(23+19)
AGCRAAAGCAGGGTGACAAAGACAAACACCCTTGTTTCTACT
>BM18_NS,1,873,39:(21+18)

AGCRAAAGCAGGGTGACAAAGAACACCCTTGTTTCTACT
>BM18_NS,1,863,49:(21+28)
AGCRAAAGCAGGGTGACAAAGAATAATAAAAAACACCCTTGTTTCTACT
>BM18_NS,1,866,43:(18+25)
AGCRAAAGCAGGGTGACAAATAAAAAACACCCTTGTTTCTACT
>BM18_NS,1,866,42:(17+25)
AGCRAAAGCAGGGTGACAATAAAAAACACCCTTGTTTCTACT
>BM18_M,1,1003,56:(31+25)
AGCRAAAGCAGGTAGATGTTGAAAGATGAGTAGTAAAAAACTACCTTGTTTCTACT
>BM18_M,1,1008,48:(28+20)
AGCRAAAGCAGGTAGATGTTGAAAGATGAAAACTACCTTGTTTCTACT
>BM18_M,1,1006,50:(28+22)
AGCRAAAGCAGGTAGATGTTGAAAGATGAAAAAACTACCTTGTTTCTACT
>BM18_M,1,1005,49:(26+23)
AGCRAAAGCAGGTAGATGTTGAAAGATAAAAAACTACCTTGTTTCTACT
>BM18_M,1,1010,43:(25+18)
AGCRAAAGCAGGTAGATGTTGAAAGAACTACCTTGTTTCTACT
>BM18_M,1,1006,47:(25+22)
AGCRAAAGCAGGTAGATGTTGAAAGAAAAAACTACCTTGTTTCTACT
>BM18_M,1,1007,42:(21+21)
AGCRAAAGCAGGTAGATGTTGAAAAACTACCTTGTTTCTACT
>BM18_M,1,1002,46:(20+26)
AGCRAAAGCAGGTAGATGTTGAGTAAAAAACTACCTTGTTTCTACT
>BM18_M,1,1003,44:(19+25)
AGCRAAAGCAGGTAGATGTAGTAAAAAACTACCTTGTTTCTACT
>BM18_M,1,1000,45:(17+28)
AGCRAAAGCAGGTAGATTGGAGTAAAAAACTACCTTGTTTCTACT
>BM18_M,1,1005,40:(17+23)
AGCRAAAGCAGGTAGATTAAAAAACTACCTTGTTTCTACT
>BM18_NA,1,1426,64:(32+32)
AGCRAAAGCAGGAGTTTAAATGAATCCAAATCGTAGTTTGTTCAAAAAACTCCTTGTTTCTACT
>BM18_NA,1,1418,63:(23+40)
AGCRAAAGCAGGAGTTTAAATGAATTGACAAGTAGTTTGTTCAAAAAACTCCTTGTTTCTACT
>BM18_NA,1,1420,58:(20+38)
AGCRAAAGCAGGAGTTTAAATGACAAGTAGTTTGTTCAAAAAACTCCTTGTTTCTACT
>BM18_NA,1,1421,55:(18+37)
AGCRAAAGCAGGAGTTTAGACAAGTAGTTTGTTCAAAAAACTCCTTGTTTCTACT
>BM18_NA,1,1438,37:(17+20)
AGCRAAAGCAGGAGTTTAAAAACTCCTTGTTTCTACT
>BM18_NP,1,1548,57:(39+18)
AGCRAAAGCAGGGTAGATAATCACTCACTGAGTGACATCAATACCCTTGTTTCTACT
>BM18_NP,1,1545,60:(39+21)
AGCRAAAGCAGGGTAGATAATCACTCACTGAGTGACATCAAAAATACCCTTGTTTCTACT
>BM18_NP,1,1547,57:(38+19)
AGCRAAAGCAGGGTAGATAATCACTCACTGAGTGACATAAATACCCTTGTTTCTACT
>BM18_NP,1,1535,67:(36+31)
AGCRAAAGCAGGGTAGATAATCACTCACTGAGTGACACAATTAAAGAAAAATACCCTTGTTTCTACT
>BM18_NP,1,1548,39:(21+18)
AGCRAAAGCAGGGTAGATAATAATACCCTTGTTTCTACT
>BM18_NP,1,1542,42:(18+24)
AGCRAAAGCAGGGTAGATAAGAAAAATACCCTTGTTTCTACT
>BM18_NP,1,1537,47:(18+29)
AGCRAAAGCAGGGTAGATAATTAAAGAAAAATACCCTTGTTTCTACT
>BM18_NP,1,1535,49:(18+31)
AGCRAAAGCAGGGTAGATACAATTAAAGAAAAATACCCTTGTTTCTACT
>BM18_NP,1,1520,62:(16+46)
AGCRAAAGCAGGGTAGATGCAGAGGAGTACGACAATTAAAGAAAAATACCCTTGTTTCTACT
>BM18_PA,1,2216,56:(38+18)
AGCRAAAGCAGGTACTGATTCAAAATGGAAGACTTTGTAAGTACCTTGTTTCTACT
>BM18_PA,1,2216,54:(36+18)
AGCRAAAGCAGGTACTGATTCAAAATGGAAGACTTTAAGTACCTTGTTTCTACT
>BM18_PA,1,2198,61:(25+36)
AGCRAAAGCAGGTACTGATTCAAAATATCCATACTGTCCAAAAAAGTACCTTGTTTCTACT
>BM18_PA,1,2215,40:(21+19)
AGCRAAAGCAGGTACTGATTCAAAGTACCTTGTTTCTACT
>BM18_PA,1,2209,43:(18+25)
AGCRAAAGCAGGTACTGATCCAAAAAAGTACCTTGTTTCTACT
>BM18_PA,1,2212,39:(17+22)
AGCRAAAGCAGGTACTGAAAAAAGTACCTTGTTTCTACT
>BM18_PB2,1,2312,64:(34+30)

AGCRAAAGCAGGTCAATTATATTCAATATGGAAAGAATAGTTTAAAAACGACCTTGTTTCTACT
>BM18_PB2,1,2313,56:(27+29)
AGCRAAAGCAGGTCAATTATATTCAATAATAGTTTAAAAACGACCTTGTTTCTACT
>BM18_PB2,1,2320,48:(26+22)
AGCRAAAGCAGGTCAATTATATTCAATAAAAACGACCTTGTTTCTACT
>BM18_PB2,1,2305,63:(26+37)
AGCRAAAGCAGGTCAATTATATTCAATAGTGTCGAATAGTTTAAAAACGACCTTGTTTCTACT
>BM18_PB2,1,2321,45:(24+21)
AGCRAAAGCAGGTCAATTATATTCAAAAACGACCTTGTTTCTACT
>BM18_PB2,1,2316,50:(24+26)
AGCRAAAGCAGGTCAATTATATTCAGTTTAAAAACGACCTTGTTTCTACT
>BM18_PB2,1,2320,41:(19+22)
AGCRAAAGCAGGTCAATTATAAAAACGACCTTGTTTCTACT
>BM18_PB2,1,2293,67:(18+49)
AGCRAAAGCAGGTCAATTATGGCCATCAATTAGTGTCGAATAGTTTAAAAACGACCTTGTTTCTACT
>BM18_PB2,1,2324,35:(17+18)
AGCRAAAGCAGGTCAATAACGACCTTGTTTCTACT
>BM18_PB1,1,2319,75:(52+23)
AGCRAAAGCAGGCAAACCATTTGAATGGATGTCAATCCGACTTTACTTTTCTTGAAAAAATGCCTTGTTTCTACT
>BM18_PB1,1,2324,62:(44+18)
AGCRAAAGCAGGCAAACCATTTGAATGGATGTCAATCCGACTTTAAATGCCTTGTTTCTACT
>BM18_PB1,1,2309,75:(42+33)
AGCRAAAGCAGGCAAACCATTTGAATGGATGTCAATCCGACTTTGTCCTTCATGAAAAAATGCCTTGTTTCTACT
>BM18_PB1,1,2324,48:(30+18)
AGCRAAAGCAGGCAAACCATTTGAATGGATAAATGCCTTGTTTCTACT
>BM18_PB1,1,2322,50:(30+20)
AGCRAAAGCAGGCAAACCATTTGAATGGATAAAAATGCCTTGTTTCTACT
>BM18_PB1,1,2318,50:(26+24)
AGCRAAAGCAGGCAAACCATTTGAATATGAAAAAATGCCTTGTTTCTACT
>BM18_PB1,1,2319,45:(22+23)
AGCRAAAGCAGGCAAACCATTTTGAAAAAATGCCTTGTTTCTACT
>BM18_PB1,1,2299,64:(21+43)
AGCRAAAGCAGGCAAACCATTTGAATTTAGCTTGTCCTTCATGAAAAAATGCCTTGTTTCTACT
>BM18_PB1,1,2309,53:(20+33)
AGCRAAAGCAGGCAAACCATTTGTCCTTCATGAAAAAATGCCTTGTTTCTACT
>BM18_PB1,1,2320,40:(18+22)
AGCRAAAGCAGGCAAACCGAAAAAATGCCTTGTTTCTACT
>BM18_PB1,1,2311,49:(18+31)
AGCRAAAGCAGGCAAACCGTCCTTCATGAAAAAATGCCTTGTTTCTACT
>BM18_PB1,1,2297,63:(18+45)
AGCRAAAGCAGGCAAACCAGTGAATTTAGCTTGTCCTTCATGAAAAAATGCCTTGTTTCTACT
>BM18_PB1,1,2317,42:(17+25)
AGCRAAAGCAGGCAAACCATGAAAAAATGCCTTGTTTCTACT
>BM18_PB1,1,2290,69:(17+52)
AGCRAAAGCAGGCAAACCAAAAGTAGTGAATTTAGCTTGTCCTTCATGAAAAAATGCCTTGTTTCTACT

Supplemental Fasta file 4. BM18 mvRNA sequences.
>BM18_PB2,1,2325,35:(18+17)
AGCRAAAGCAGGTCAATTACGACCTTGTTTCTACT
>BM18_M,1,1010,35:(17+18)
AGCRAAAGCAGGTAGATAACTACCTTGTTTCTACT
>BM18_M,1,1009,36:(17+19)
AGCRAAAGCAGGTAGATAAACTACCTTGTTTCTACT
>BM18_PB2,1,2324,36:(18+18)
AGCRAAAGCAGGTCAATTAACGACCTTGTTTCTACT
>BM18_NS,1,872,36:(17+19)
AGCRAAAGCAGGGTGACAAACACCCTTGTTTCTACT
>BM18_NP,1,1548,34:(16+18)
AGCRAAAGCAGGGTAGAATACCCTTGTTTCTACT
>BM18_NP,1,1547,37:(18+19)
AGCRAAAGCAGGGTAGATAAATACCCTTGTTTCTACT
>BM18_NP,1,1547,35:(16+19)
AGCRAAAGCAGGGTAGAAATACCCTTGTTTCTACT
>BM18_HA,1,1762,36:(19+17)
AGCRAAAGCAGGGGAAAATACACCCTTGTTTCTACT
>BM18_NS,1,873,35:(17+18)
AGCRAAAGCAGGGTGACAACACCCTTGTTTCTACT
>BM18_NP,1,1545,39:(18+21)
AGCRAAAGCAGGGTAGATAAAAATACCCTTGTTTCTACT
>BM18_HA,1,1752,44:(17+27)
AGCRAAAGCAGGGGAAAATAAGGAAAAACACCCTTGTTTCTACT
>BM18_PB2,1,2323,37:(18+19)
AGCRAAAGCAGGTCAATTAAACGACCTTGTTTCTACT
>BM18_NS,1,871,37:(17+20)
AGCRAAAGCAGGGTGACAAAACACCCTTGTTTCTACT
>BM18_HA,1,1761,37:(19+18)
AGCRAAAGCAGGGGAAAATAACACCCTTGTTTCTACT
>BM18_NP,1,1546,36:(16+20)
AGCRAAAGCAGGGTAGAAAATACCCTTGTTTCTACT
>BM18_HA,1,1757,41:(19+22)
AGCRAAAGCAGGGGAAAATGAAAAACACCCTTGTTTCTACT
>BM18_NS,1,869,39:(17+22)
AGCRAAAGCAGGGTGACAAAAAACACCCTTGTTTCTACT
>BM18_NP,1,1546,38:(18+20)
AGCRAAAGCAGGGTAGATAAAATACCCTTGTTTCTACT
>BM18_PB2,1,2319,39:(16+23)
AGCRAAAGCAGGTCAATTAAAAACGACCTTGTTTCTACT
>BM18_M,1,1008,37:(17+20)
AGCRAAAGCAGGTAGATAAAACTACCTTGTTTCTACT
>BM18_HA,1,1760,38:(19+19)
AGCRAAAGCAGGGGAAAATAAACACCCTTGTTTCTACT
>BM18_NP,1,1540,43:(17+26)
AGCRAAAGCAGGGTAGATAAAGAAAAATACCCTTGTTTCTACT
>BM18_M,1,1005,39:(16+23)
AGCRAAAGCAGGTAGATAAAAAACTACCTTGTTTCTACT
>BM18_PB1,1,2324,36:(18+18)
AGCRAAAGCAGGCAAACCAAATGCCTTGTTTCTACT
>BM18_NP,1,1544,40:(18+22)
AGCRAAAGCAGGGTAGATGAAAAATACCCTTGTTTCTACT
>BM18_NP,1,1549,35:(18+17)
AGCRAAAGCAGGGTAGATATACCCTTGTTTCTACT
>BM18_M,1,1011,34:(17+17)
AGCRAAAGCAGGTAGATACTACCTTGTTTCTACT
>BM18_PB1,1,2323,37:(18+19)
AGCRAAAGCAGGCAAACCAAAATGCCTTGTTTCTACT
>BM18_HA,1,1758,37:(16+21)
AGCRAAAGCAGGGGAAAAAAACACCCTTGTTTCTACT
>BM18_NP,1,1543,41:(18+23)
AGCRAAAGCAGGGTAGATAGAAAAATACCCTTGTTTCTACT
>BM18_NS,1,874,34:(17+17)
AGCRAAAGCAGGGTGACACACCCTTGTTTCTACT
>BM18_PA,1,2216,34:(16+18)
AGCRAAAGCAGGTACTAAGTACCTTGTTTCTACT
>BM18_HA,1,1756,42:(19+23)
AGCRAAAGCAGGGGAAAATGGAAAAACACCCTTGTTTCTACT
>BM18_PB2,1,2315,44:(17+27)

AGCRAAAGCAGGTCAATTAGTTTAAAAACGACCTTGTTTCTACT
>BM18_NS,1,870,38:(17+21)
AGCRAAAGCAGGGTGACAAAAACACCCTTGTTTCTACT
>BM18_PB2,1,2322,38:(18+20)
AGCRAAAGCAGGTCAATTAAAACGACCTTGTTTCTACT
>BM18_PB1,1,2325,35:(18+17)
AGCRAAAGCAGGCAAACCAATGCCTTGTTTCTACT
>BM18_HA,1,1758,38:(17+21)
AGCRAAAGCAGGGGAAAAAAAACACCCTTGTTTCTACT
>BM18_HA,1,1754,42:(17+25)
AGCRAAAGCAGGGGAAAAAGGAAAAACACCCTTGTTTCTACT
>BM18_HA,1,1759,39:(19+20)
AGCRAAAGCAGGGGAAAATAAAACACCCTTGTTTCTACT
>BM18_NA,1,1439,36:(17+19)
AGCRAAAGCAGGAGTTTAAAACTCCTTGTTTCTACT
>BM18_M,1,1007,38:(17+21)
AGCRAAAGCAGGTAGATAAAAACTACCTTGTTTCTACT
>BM18_NA,1,1437,38:(17+21)
AGCRAAAGCAGGAGTTTAAAAAACTCCTTGTTTCTACT
>BM18_PB1,1,2321,39:(18+21)
AGCRAAAGCAGGCAAACCAAAAAATGCCTTGTTTCTACT
>BM18_NS,1,867,47:(23+24)
AGCRAAAGCAGGGTGACAAAGACATAAAAAACACCCTTGTTTCTACT
>BM18_NP,1,1532,62:(28+34)
AGCRAAAGCAGGGTAGATAATCACTCACACGACAATTAAAGAAAAATACCCTTGTTTCTACT
>BM18_NP,1,1541,41:(16+25)
AGCRAAAGCAGGGTAGAAAGAAAAATACCCTTGTTTCTACT
>BM18_PB1,1,2322,38:(18+20)
AGCRAAAGCAGGCAAACCAAAAATGCCTTGTTTCTACT
>BM18_NP,1,1531,52:(17+35)
AGCRAAAGCAGGGTAGATACGACAATTAAAGAAAAATACCCTTGTTTCTACT
>BM18_PA,1,2212,48:(26+22)
AGCRAAAGCAGGTACTGATTCAAAATAAAAAAGTACCTTGTTTCTACT
>BM18_HA,1,1752,59:(32+27)
AGCRAAAGCAGGGGAAAATAAAAACAACCAAAATAAGGAAAAACACCCTTGTTTCTACT
>BM18_HA,1,1754,41:(16+25)
AGCRAAAGCAGGGGAAAAGGAAAAACACCCTTGTTTCTACT
>BM18_PA,1,2211,43:(20+23)
AGCRAAAGCAGGTACTGATTCAAAAAAGTACCTTGTTTCTACT
>BM18_HA,1,1755,43:(19+24)
AGCRAAAGCAGGGGAAAATAGGAAAAACACCCTTGTTTCTACT
>BM18_HA,1,1737,60:(18+42)
AGCRAAAGCAGGGGAAAATAGGATTTCAGAAATATAAGGAAAAACACCCTTGTTTCTACT
>BM18_PB2,1,2313,47:(18+29)
AGCRAAAGCAGGTCAATTAATAGTTTAAAAACGACCTTGTTTCTACT
>BM18_NS,1,864,50:(23+27)
AGCRAAAGCAGGGTGACAAAGACATAATAAAAAACACCCTTGTTTCTACT
>BM18_NP,1,1546,56:(36+20)
AGCRAAAGCAGGGTAGATAATCACTCACTGAGTGACAAAATACCCTTGTTTCTACT
>BM18_NP,1,1535,59:(28+31)
AGCRAAAGCAGGGTAGATAATCACTCACACAATTAAAGAAAAATACCCTTGTTTCTACT
>BM18_PA,1,2212,44:(22+22)
AGCRAAAGCAGGTACTGATTCAAAAAAAGTACCTTGTTTCTACT
>BM18_PA,1,2215,40:(21+19)
AGCRAAAGCAGGTACTGATTCAAAGTACCTTGTTTCTACT
>BM18_PA,1,2208,47:(21+26)
AGCRAAAGCAGGTACTGATTCGTCCAAAAAAGTACCTTGTTTCTACT
>BM18_PA,1,2215,35:(16+19)
AGCRAAAGCAGGTACTAAAGTACCTTGTTTCTACT
>BM18_PB2,1,2313,53:(24+29)
AGCRAAAGCAGGTCAATTATATTCAATAGTTTAAAAACGACCTTGTTTCTACT
>BM18_PB2,1,2314,46:(18+28)
AGCRAAAGCAGGTCAATTATAGTTTAAAAACGACCTTGTTTCTACT
>BM18_PB2,1,2323,36:(17+19)
AGCRAAAGCAGGTCAATAAACGACCTTGTTTCTACT
>BM18_HA,1,1748,61:(30+31)
AGCRAAAGCAGGGGAAAATAAAAACAACCAAAATATAAGGAAAAACACCCTTGTTTCTACT
>BM18_HA,1,1738,66:(25+41)
AGCRAAAGCAGGGGAAAATAAAAACAGGATTTCAGAAATATAAGGAAAAACACCCTTGTTTCTACT
>BM18_HA,1,1758,39:(18+21)

```
AGCRAAAGCAGGGGAAAAAAAAACACCCTTGTTTCTACT
>BM18_HA,1,1757,39:(17+22)
AGCRAAAGCAGGGGAAAGAAAAACACCCTTGTTTCTACT
>BM18_HA,1,1738,58:(17+41)
AGCRAAAGCAGGGGAAAAGGATTTCAGAAATATAAGGAAAAACACCCTTGTTTCTACT
>BM18_NS,1,866,55:(30+25)
AGCRAAAGCAGGGTGACAAAGACATAATGGAATAAAAAACACCCTTGTTTCTACT
>BM18_M,1,1011,35:(18+17)
AGCRAAAGCAGGTAGATGACTACCTTGTTTCTACT
>BM18_NA,1,1428,57:(27+30)
AGCRAAAGCAGGAGTTTAAATGAATCCAGTTTGTTCAAAAAACTCCTTGTTTCTACT
>BM18_NA,1,1427,47:(16+31)
AGCRAAAGCAGGAGTTTAGTTTGTTCAAAAAACTCCTTGTTTCTACT
>BM18_NP,1,1548,57:(39+18)
AGCRAAAGCAGGGTAGATAATCACTCACTGAGTGACATCAATACCCTTGTTTCTACT
>BM18_NP,1,1535,57:(26+31)
AGCRAAAGCAGGGTAGATAATCACTCACAATTAAAGAAAAATACCCTTGTTTCTACT
>BM18_PA,1,2207,52:(25+27)
AGCRAAAGCAGGTACTGATTCAAAATGTCCAAAAAAGTACCTTGTTTCTACT
>BM18_PA,1,2215,36:(17+19)
AGCRAAAGCAGGTACTGAAAGTACCTTGTTTCTACT
>BM18_PA,1,2214,36:(16+20)
AGCRAAAGCAGGTACTAAAAGTACCTTGTTTCTACT
>BM18_PB2,1,2320,48:(26+22)
AGCRAAAGCAGGTCAATTATATTCAATAAAAACGACCTTGTTTCTACT
>BM18_PB1,1,2321,43:(22+21)
AGCRAAAGCAGGCAAACCATTTAAAAAATGCCTTGTTTCTACT
>BM18_PB1,1,2319,44:(21+23)
AGCRAAAGCAGGCAAACCATTTGAAAAAATGCCTTGTTTCTACT
>BM18_PB1,1,2320,39:(17+22)
AGCRAAAGCAGGCAAACGAAAAAATGCCTTGTTTCTACT
>BM18_HA,1,1752,45:(18+27)
AGCRAAAGCAGGGGAAAAATAAGGAAAAACACCCTTGTTTCTACT
>BM18_HA,1,1731,66:(18+48)
AGCRAAAGCAGGGGAAAATGAGATTAGGATTTCAGAAATATAAGGAAAAACACCCTTGTTTCTACT
>BM18_NS,1,869,52:(30+22)
AGCRAAAGCAGGGTGACAAAGACATAATGGAAAAAACACCCTTGTTTCTACT
>BM18_NS,1,872,40:(21+19)
AGCRAAAGCAGGGTGACAAAGAAACACCCTTGTTTCTACT
>BM18_M,1,1004,41:(17+24)
AGCRAAAGCAGGTAGATGTAAAAAACTACCTTGTTTCTACT
>BM18_NA,1,1433,53:(28+25)
AGCRAAAGCAGGAGTTTAAATGAATCCAGTTCAAAAAACTCCTTGTTTCTACT
>BM18_NP,1,1544,61:(39+22)
AGCRAAAGCAGGGTAGATAATCACTCACTGAGTGACATCGAAAAATACCCTTGTTTCTACT
>BM18_NP,1,1550,37:(21+16)
AGCRAAAGCAGGGTAGATAATTACCCTTGTTTCTACT
>BM18_NP,1,1529,55:(18+37)
AGCRAAAGCAGGGTAGATAGTACGACAATTAAAGAAAAATACCCTTGTTTCTACT
>BM18_NP,1,1544,38:(16+22)
AGCRAAAGCAGGGTAGGAAAAATACCCTTGTTTCTACT
>BM18_PA,1,2207,64:(37+27)
AGCRAAAGCAGGTACTGATTCAAAATGGAAGACTTTGTGTCCAAAAAAGTACCTTGTTTCTACT
>BM18_PA,1,2214,41:(21+20)
AGCRAAAGCAGGTACTGATTCAAAAGTACCTTGTTTCTACT
>BM18_PA,1,2217,33:(16+17)
AGCRAAAGCAGGTACTAGTACCTTGTTTCTACT
>BM18_PB2,1,2324,35:(17+18)
AGCRAAAGCAGGTCAATAACGACCTTGTTTCTACT
>BM18_PB1,1,2294,74:(26+48)
AGCRAAAGCAGGCAAACCATTTGAATAGTAGTGAATTTAGCTTGTCCTTCATGAAAAAATGCCTTGTTTCTACT
>BM18_PB1,1,2326,36:(20+16)
AGCRAAAGCAGGCAAACCATATGCCTTGTTTCTACT
>BM18_PB1,1,2320,40:(18+22)
AGCRAAAGCAGGCAAACCGAAAAAATGCCTTGTTTCTACT
>BM18_PB1,1,2324,35:(17+18)
AGCRAAAGCAGGCAAACAAATGCCTTGTTTCTACT
>BM18_PB1,1,2323,36:(17+19)
AGCRAAAGCAGGCAAACAAAATGCCTTGTTTCTACT
>BM18_HA,1,1737,75:(33+42)
```

AGCRAAAGCAGGGGAAAATAAAAACAACCAAAATAGGATTTCAGAAATATAAGGAAAAACACCCTTGTTTCTACT
>BM18_HA,1,1757,43:(21+22)
AGCRAAAGCAGGGGAAAATAAGAAAAACACCCTTGTTTCTACT
>BM18_NS,1,868,63:(40+23)
AGCRAAAGCAGGGTGACAAAGACATAATGGATTCTAACACTAAAAAACACCCTTGTTTCTACT
>BM18_NS,1,871,45:(25+20)
AGCRAAAGCAGGGTGACAAAGACATAAAACACCCTTGTTTCTACT
>BM18_NS,1,862,53:(24+29)
AGCRAAAGCAGGGTGACAAAGACATAATAATAAAAAACACCCTTGTTTCTACT
>BM18_NS,1,873,39:(21+18)
AGCRAAAGCAGGGTGACAAAGAACACCCTTGTTTCTACT
>BM18_NS,1,869,43:(21+22)
AGCRAAAGCAGGGTGACAAAGAAAAAACACCCTTGTTTCTACT
>BM18_NS,1,837,73:(19+54)
AGCRAAAGCAGGGTGACAAAAGAACTTTCTCGTTTCAGCTTATTTAATAATAAAAAACACCCTTGTTTCTACT
>BM18_NS,1,875,33:(17+16)
AGCRAAAGCAGGGTGACCACCCTTGTTTCTACT
>BM18_M,1,1006,69:(47+22)
AGCRAAAGCAGGTAGATGTTGAAAGATGAGTCTTCTAACCGAGGTCGAAAAAACTACCTTGTTTCTACT
>BM18_M,1,1010,45:(27+18)
AGCRAAAGCAGGTAGATGTTGAAAGATAACTACCTTGTTTCTACT
>BM18_M,1,1000,54:(26+28)
AGCRAAAGCAGGTAGATGTTGAAAGATGGAGTAAAAAACTACCTTGTTTCTACT
>BM18_M,1,1002,43:(17+26)
AGCRAAAGCAGGTAGATGAGTAAAAAACTACCTTGTTTCTACT
>BM18_NA,1,1429,56:(27+29)
AGCRAAAGCAGGAGTTTAAATGAATCCGTTTGTTCAAAAAACTCCTTGTTTCTACT
>BM18_NA,1,1438,36:(16+20)
AGCRAAAGCAGGAGTTAAAAACTCCTTGTTTCTACT
>BM18_NP,1,1548,69:(51+18)
AGCRAAAGCAGGGTAGATAATCACTCACTGAGTGACATCAAAATCATGGCGAATACCCTTGTTTCTACT
>BM18_NP,1,1541,64:(39+25)
AGCRAAAGCAGGGTAGATAATCACTCACTGAGTGACATCAAAGAAAAATACCCTTGTTTCTACT
>BM18_NP,1,1547,55:(36+19)
AGCRAAAGCAGGGTAGATAATCACTCACTGAGTGACAAATACCCTTGTTTCTACT
>BM18_NP,1,1545,42:(21+21)
AGCRAAAGCAGGGTAGATAATAAAAATACCCTTGTTTCTACT
>BM18_NP,1,1550,34:(18+16)
AGCRAAAGCAGGGTAGATTACCCTTGTTTCTACT
>BM18_NP,1,1542,42:(18+24)
AGCRAAAGCAGGGTAGATAAGAAAAATACCCTTGTTTCTACT
>BM18_NP,1,1537,47:(18+29)
AGCRAAAGCAGGGTAGATAATTAAAGAAAAATACCCTTGTTTCTACT
>BM18_NP,1,1535,49:(18+31)
AGCRAAAGCAGGGTAGATACAATTAAAGAAAAATACCCTTGTTTCTACT
>BM18_NP,1,1539,43:(16+27)
AGCRAAAGCAGGGTAGTTAAAGAAAAATACCCTTGTTTCTACT
>BM18_PA,1,2208,57:(31+26)
AGCRAAAGCAGGTACTGATTCAAAATGGAAGGTCCAAAAAAGTACCTTGTTTCTACT
>BM18_PA,1,2216,39:(21+18)
AGCRAAAGCAGGTACTGATTCAAGTACCTTGTTTCTACT
>BM18_PA,1,2213,41:(20+21)
AGCRAAAGCAGGTACTGATTAAAAAGTACCTTGTTTCTACT
>BM18_PA,1,2217,34:(17+17)
AGCRAAAGCAGGTACTGAGTACCTTGTTTCTACT
>BM18_PA,1,2213,37:(16+21)
AGCRAAAGCAGGTACTAAAAAGTACCTTGTTTCTACT
>BM18_PB2,1,2323,48:(29+19)
AGCRAAAGCAGGTCAATTATATTCAATATAAACGACCTTGTTTCTACT
>BM18_PB2,1,2306,61:(25+36)
AGCRAAAGCAGGTCAATTATATTCAAGTGTCGAATAGTTTAAAAACGACCTTGTTTCTACT
>BM18_PB2,1,2314,48:(20+28)
AGCRAAAGCAGGTCAATTATATAGTTTAAAAACGACCTTGTTTCTACT
>BM18_PB2,1,2326,34:(18+16)
AGCRAAAGCAGGTCAATTCGACCTTGTTTCTACT
>BM18_PB2,1,2293,67:(18+49)
AGCRAAAGCAGGTCAATTATGGCCATCAATTAGTGTCGAATAGTTTAAAAACGACCTTGTTTCTACT
>BM18_PB2,1,2325,34:(17+17)
AGCRAAAGCAGGTCAATACGACCTTGTTTCTACT
>BM18_PB2,1,2308,51:(17+34)

AGCRAAAGCAGGTCAATTGTCGAATAGTTTAAAAACGACCTTGTTTCTACT
>BM18_PB2,1,2320,38:(16+22)
AGCRAAAGCAGGTCAATAAAAACGACCTTGTTTCTACT
>BM18_PB1,1,2314,73:(45+28)
AGCRAAAGCAGGCAAACCATTTGAATGGATGTCAATCCGACTTTACTTCATGAAAAAATGCCTTGTTTCTACT
>BM18_PB1,1,2323,49:(30+19)
AGCRAAAGCAGGCAAACCATTTGAATGGATAAAATGCCTTGTTTCTACT
>BM18_PB1,1,2321,47:(26+21)
AGCRAAAGCAGGCAAACCATTTGAATAAAAAATGCCTTGTTTCTACT
>BM18_PB1,1,2320,41:(19+22)
AGCRAAAGCAGGCAAACCAGAAAAAATGCCTTGTTTCTACT
>BM18_PB1,1,2325,34:(17+17)
AGCRAAAGCAGGCAAACAATGCCTTGTTTCTACT
>BM18_PB1,1,2317,42:(17+25)
AGCRAAAGCAGGCAAACCATGAAAAAATGCCTTGTTTCTACT

Supplemental script – Python script for sliding window analysis

```
#Influenza virus RNA polymerase simulation script that searches for t-loops and checks up/down stream for intermol bp
#Uses Python 3.8, biopython, openpyxl, and Vienna RNA 2.47; side packages were installed with Anaconda3
#By AJ te Velthuis, Sept 2020

import sys
sys.path.append("/users/USER/opt/anaconda3/lib/python3.8/site-packages")

##input sequence. Paste sequence between quotation marks below.

RNA = "PASTE SEQUENCE HERE"

Name ="Template"

##output bubbles in txt file
f= open("deltaGvalues.txt","w+")

##specify polymerase properties

Footprint = 20

##specify NP footprint

NP = 24

TloopDuplex = 48

Duplex = int(TloopDuplex / 2)

##specify window and other comparisons

Uloop = "&" #Use & for co-fold to compute long-range interactions between upstream and downstream sequences.

Swindow = 1  #size of sliding window


###########################################

##invert input sequence to start at 3' end

NegRNA = RNA[::-1]

Length  = len(NegRNA)

##########################################

##polymerase bubble properties

Bubble = Footprint + Duplex

##iteration start point of simulation; start at nt 2 otherwise downstream sequence is empty for co-fold
i = 1
```

```
##end bubble sequence and add 1, because sequence count starts at 0

End = int((Length - Footprint + 1) / Swindow)


##find polymerase bubble sequence; allow for small 3' part to emerge, but
##then cap how long the emerging sequence can be by assuming that every 24 nt will be bound by NP
##unclear if NP binds in chunks or progressively. Assume that at least 24 nt are needed based on data from Ortin lab.
##stops 1 nt from end to avoid having no sequence in cofold

for i in range(1, End-1):

    if i <= NP:
        Upstream = i
        Downstream = 0
    else:
        Upstream = NP
        Downstream = i-NP

    #find upstream and downstream sequence of the bubble for intermolecular folding check

    Ahead = NegRNA[Footprint+i:Footprint+NP+i]

    Aheadinv = Ahead[::-1]

    #currently only takes 1 nt of downstream as 0 gives an error in cofold.
    Down = NegRNA[Downstream:i]

    Downinv = Down[::-1]

    ##find the two duplex ends and invert them so they are 5' to 3'

    if i <= Duplex:
        Prime3 = NegRNA[0:Upstream]
    else:
        Prime3 = NegRNA[i-Duplex:i]

    Prime3inv = Prime3[::-1]

    if i <= End:
        Prime5 = NegRNA[Footprint+i:Bubble+i]
    else:
        Prime5 = NegRNA[Footprint+i:Length]

    Prime5inv = Prime5[::-1]

    #compute A/U content of nucleotides in active site. Assume window of 6 before and after active site. Various print options are inactivated, but can be used for checking if script works.

    ActiveSite = i + 16

    #print("location of bubble is ", i+1)

    #print("3 prime end 3' to 5' is ", Prime3)
```

```
    #print("5 prime end 5' to 3' is ", Prime5inv)

    ActiveSiteSeqUp = NegRNA[ActiveSite-1:ActiveSite+4]

    ActiveSiteSeqDown = NegRNA[ActiveSite-5:ActiveSite]

    #seq_up_list = list(ActiveSiteSeqUp)
    seq_up_list = list(ActiveSiteSeqDown)
    at_count_up = seq_up_list.count("a") + seq_up_list.count ("t") + seq_up_list.count("A") + seq_up_list.count ("T") + seq_up_list.count ("u") + seq_up_list.count ("U")
    at_frac_up = float(at_count_up)/5
    total_at_up = 100 * at_frac_up

##saving the A/U content is inactivated below
#print(total_at_up)
    #f.write("%f\n" % (total_at_up))

    ##Vienna RNA package for RNA structure prediction
    import RNA

    Test = Prime3 + Uloop + Prime5

    NegTest = Test[::-1]

    #Output = (">"+Name+"_polymerase_bubble_number_%d\n" % (i+1))

    ##To write bubble sequence to .txt file
    #f.write(Output)
    #f.write(NegTest)
    #f.write("\r\n")

    #use duplex fold to compute t-loop from Vienna package because it ignores intermol bp
    duplex = RNA.duplexfold(Prime5inv, Prime3inv)

    #print("%s\n%s [%6.2f]" % (NegTest, duplex.structure, duplex.energy))
    ##print("%6.2f" % (duplex.energy))

    #use cofold from Vienna package to check for bp in sequence upstream and downstream of t-loop. Various options can be checked separately, including just upstream seq, just downstream seq, or both seq
    Other = Aheadinv + Uloop + Downinv
    #Other = Aheadinv
    #Other = Downinv

    (ss, mfe_dimer) = RNA.cofold(Other)
    #print("%s\n%s  [%6.2f]" % (Other, ss, mfe_dimer))

    DDeltaG = duplex.energy - mfe_dimer ##compute DeltaDeltaG

    ##check if deltaG of t-loop is lower than deltaG of other structures
    if duplex.energy >= mfe_dimer:
        DeltaG = mfe_dimer * -1
    #elif duplex.energy >= 0:
        #DeltaG = 0
    else:
        #DeltaG = duplex.energy
```

```
        DeltaG = duplex.energy

    #DeltaG = mfe_dimer
    #DeltaG = duplex.energy

    #print(DeltaG)
    #print(DDeltaG)
    #print(duplex.energy)

    #print("%s\n%s [ %6.2f ]" % (NegTest, ss, mfe))

    ##to write deltaG values to .txt file. Various options are available depending on what is being analyzed.
    f.write("%f\n" % (duplex.energy))
    #f.write("%f\n" % (DeltaG))
    #f.write("%f\n" % (mfe_dimer))

    #print(NegTest)
    #print("-----")

    i =+ Swindow

####################################################

##close .txt file that deltaG values were written to
f.close()
print ("Done %s" % (Name))
```
